# HSP70 and HSP90 in Cancer: Cytosolic, Endoplasmic Reticulum and Mitochondrial Chaperones of Tumorigenesis

**DOI:** 10.3389/fonc.2022.829520

**Published:** 2022-01-21

**Authors:** Zarema Albakova, Yana Mangasarova, Akhmet Albakov, Liliya Gorenkova

**Affiliations:** ^1^ Department of Biology, Lomonosov Moscow State University, Moscow, Russia; ^2^ Chokan Limited Liability Partnership (LLP), Almaty, Kazakhstan; ^3^ National Research Center for Hematology, Moscow, Russia; ^4^ Department of Innovation, Almaty, Kazakhstan

**Keywords:** heat shock proteins, HSP70, HSP90, GRP78, GRP94, TRAP1, mortalin, cancer

## Abstract

HSP70 and HSP90 are two powerful chaperone machineries involved in survival and proliferation of tumor cells. Residing in various cellular compartments, HSP70 and HSP90 perform specific functions. Concurrently, HSP70 and HSP90 homologs may also translocate from their primary site under various stress conditions. Herein, we address the current literature on the role of HSP70 and HSP90 chaperone networks in cancer. The goal is to provide a comprehensive review on the functions of cytosolic, mitochondrial and endoplasmic reticulum HSP70 and HSP90 homologs in cancer. Given that high expression of HSP70 and HSP90 enhances tumor development and associates with tumor aggressiveness, further understanding of HSP70 and HSP90 chaperone networks may provide clues for the discoveries of novel anti-cancer therapies.

## Introduction

Heat shock protein 70 kDa (HSP70) and HSP90 are two powerful ATPase-dependent chaperone machineries involved in protein folding, degradation, maturation of client proteins and protein trafficking (1–4). Over the last decade, HSP90 and HSP70 have gained a lot of attention due to their critical roles in cancer (5–7). Currently, a large number of preclinical and clinical studies assess various ways of exploiting HSP70 and HSP90 machineries for the discovery of effective anti-cancer therapies (8).

HSP90 family is composed of four members: two in cytosol (HSP90AA1&HSP90AB1), one in endoplasmic reticulum (ER) (GRP94/HSP90B1) and one in mitochondria (TRAP1) (9, 10). Even though conformational states are conserved in all HSP90 members, each HSP90 homolog has its own kinetics and equilibria, suggesting specific functions in the relevant subcellular compartment (11). Сytosolic HSP90 members require co-chaperones for their functional cycles, though no co-chaperones have been yet identified for mitochondrial and ER HSP90 chaperones (11, 12).

HSP70 family is composed of 13 members and the most well-studied are: cytosolic HSP70/HSPA1A and HSC70/HSPA8, mitochondrial HSP70 homolog known as mortalin/glucose-regulated protein 75 (GRP75), and an ER HSP70 member- HSPA5/GRP78 also known as binding immunoglobulin protein (BiP) (10, 13). Similar to HSP90, HSP70s require co-chaperones for the regulation of their functional cycles (5).

HSP90 and HSP70 play essential role in proteome homeostasis (14). HSP70 binds to virtually all unfolded or misfolded proteins while HSP90 interacts with specific set of clients [reviewed in (13, 15, 16)] (17). Both chaperones undergo conformational changes to facilitate the binding and release of client proteins (13, 17). HSP70 is composed of N-terminal nucleotide-binding domain (NBD) and C –terminal substrate-binding domain (SBD), comprising an α-helical lid (SBDα) and a β-sandwich core (SBDβ) (13). HSP90 is composed of three domains, such as N-terminal (NTD) and middle domains (MD), required for ATP binding and hydrolysis, and C-terminal domain (CTD), which is essential for dimerization (18). HSP70 typically acts early in the folding process, while HSP90 functions later (17). HSP70 functional cycle is tightly regulated by HSP40 co-chaperone and nucleotide-exchange factors (NEFs) (13). Upon release from HSP70, newly synthesized polypeptides will either fold spontaneously or will be transferred to HSP90 for further folding or targeted for proteasomal degradation (5, 13). The function of HSP90 and its co-chaperones is also regulated by various post-translational modifications. Acetylation and phosphorylation may affect ATPase activity, client and co-chaperone binding (15). Furthermore, HSP90 can also be ubiquitinated by the C- terminus of HSC70-interacting protein (CHIP) [reviewed in (15)]. CHIP is an E3 ubiquitin - protein ligase, which binds to the C terminal EEVD motif of HSP70 and HSP90 chaperones *via* its tetratricopeptide repeat (TPR) domain (13, 19). Additionally, HSP90 and HSP70 folding activity can also be affected by reactive aldehydes generated from lipid peroxidation (20).

HSP70 and HSP90 molecular chaperones collaborate with each other in the process of protein remodeling. Several studies have demonstrated that HSP70, HSP90 and co-chaperones regulate the tumor suppressor protein p53 (21, 22). In a recent study, Boysen and colleagues have reported that stress-inducible HSP70 isoform (HSPA1A) and DNAJB1 co-chaperone unfold the p53 DNA binding domain (DBD) while HSP90 protects the p53 DBD from unfolding (23). Similar HSP90 and HSP70 functional antagonism has also been observed for other client proteins. Wang and colleagues reported that HSP70 binds and inactivates the glucocorticoid receptor (GR) ligand-binding domain and loads it onto HSP90 *via* HSP70 and HSP90 organizing protein (HOP), leading to the formation of GR-maturation complex (17).

**Figure 1:**
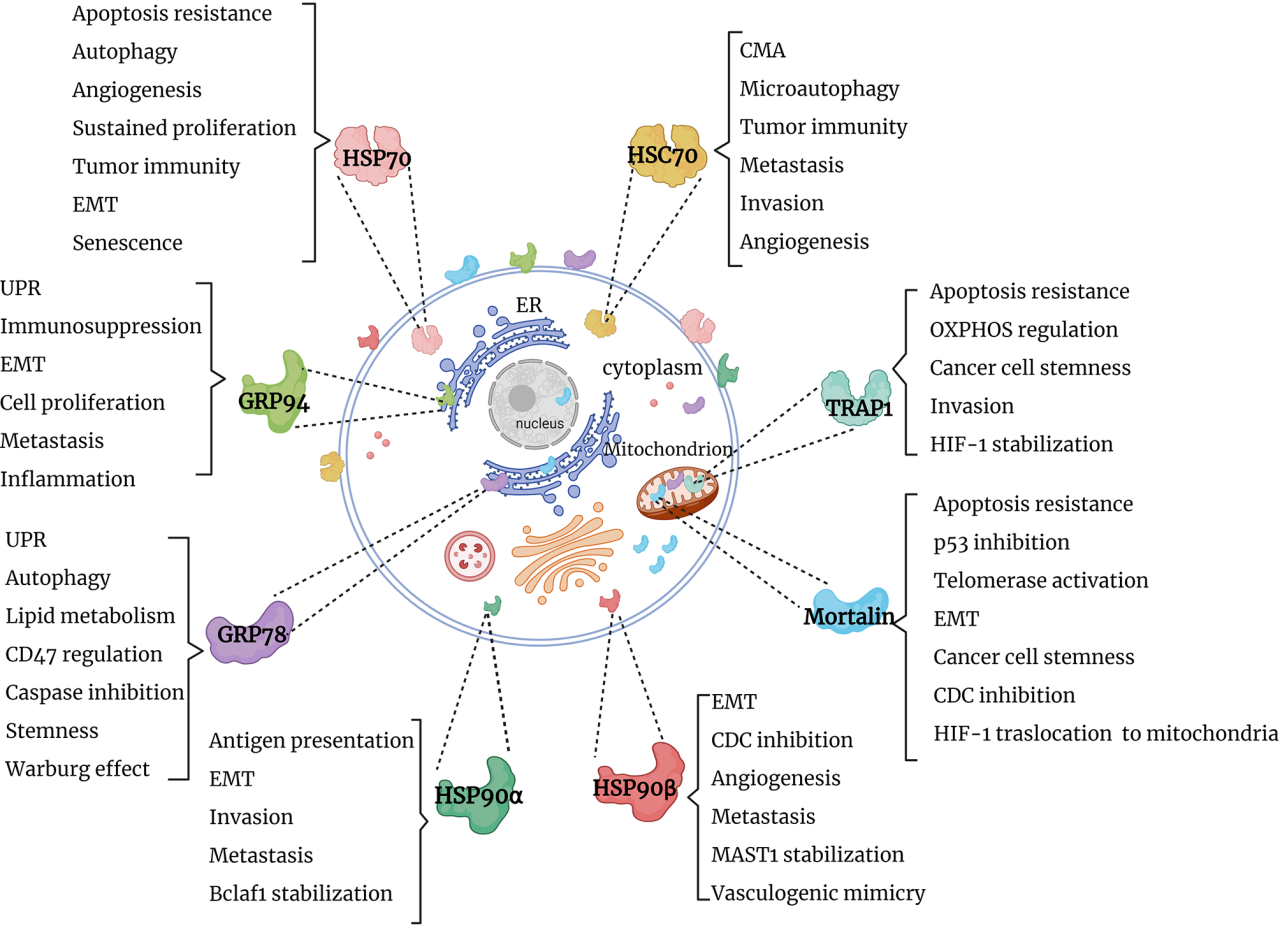
Graphical summary of HSP90 and HSP70 functions in cancer. HSP90 and HSP70 homologs are distributed in cytosol, nucleus, ER and mitochondria, where they perform specific functions, supporting tumor survival and growth. EMT, epithelial-mesenchymal transition; UPR, unfolded protein response; OXPHOS, oxidative phosphorylation; CMA, chaperone-mediated autophagy; CDC, complement-dependent cytotoxicity; Bclaf1, B-cell lymphoma 2 -associated transcription factor 1; HIF-1, hypoxia-inducible factor 1; MAST1, microtubule-associated serine/threonine kinase 1.

Several research groups reported the presence of HSPs in extracellular milieu. Specifically, HSP70 family members (HSP70/HSPA1A and mortalin), HSP90 family members (GRP78, HSP90α and HSP90β), HSP60 and HSP27 were identified on the cell surface of tumor cells (24–26). Along this line, the majority of HSP70 and HSP90 members and their co-chaperones were identified in extracellular vesicles derived from various liquid biopsies of cancer patients (27–31) [reviewed in (8)]. Furthermore, HSP70 and HSP90 family members and co-chaperones have been shown to be released by immune cells in extracellular vesicles (8, 32–36). It is also worth mentioning that extracellular HSP70 and HSP90 homologs modulate various components of the immune system [reviewed in (37)]. Currently, various studies are aimed at exploiting extracellular HSPs as a diagnostic tool and as therapeutic targets (8, 38–43). This review will focus on functions of the cytosolic, mitochondrial and ER members of HSP70 and HSP90 chaperone machineries in cancer (**Figure 1**). Further understanding of HSP70 and HSP90 functions may provide clues on their roles in cancer progression and open new perspectives for the development of novel anti-cancer therapies.

## Cytosolic HSP90 and HSP70 in Cancer

### HSP90α/HSP90AA1 and HSP90β/HSP90AB1

HSP90α and HSP90β are the two main cytosolic HSP90 isoforms encoded by two different genes, namely *HSP90AA1* and *HSP90AB1*, respectively (44, 45). HSP90α is induced upon inflammation, proteotoxic and other stress conditions, whereas HSP90β is constitutively expressed (44, 45). Even though the two isoforms share a high degree of identity (85%), they have distinct functions (44, 45). Taipale and colleagues predicted that HSP90 interacts with 7% of the transcription factors, 60% of the protein kinases and 30% of mammalian E3-ubiquitin ligases in the human genome (46). In this regard, Prince and collaborators compared relative interaction strength for both isoforms and demonstrated that HSP90α binds hypoxia-inducible factor 1α (HIF-1α) with higher relative interaction strength than to the heat shock factor 1 (HSF-1) (45). By contrast, HSP90β had higher relative interaction strength towards HSF-1 than to HIF-1α (45). This was further supported by the finding that HSP90α-knockout cells are more prone to hypoxia-induced cell death, while addition of purified recombinant extracellular HSP90α prevented cell death under hypoxia (47). Along this line, downregulation of HIF-1α resulted in decreased HSP90α expression in metastatic breast cancer cells (48).

Recently, Ono and colleagues have shown that triple deletion of HSP90α/β and CDC37 co-chaperone reduced epithelial-mesenchymal transition (EMT), attenuated extracellular vesicle (EV)-driven tumorsphere formation and EV-driven macrophage M2 polarization in metastatic oral cancer (49). Results also showed that a high HSP90α-positive cancer cell rate correlated with high-grade tumors, whereas HSP90β-positive cancer cell rate associated with low-grade tumors (49). Furthermore, in contrast to low-grade tumors, HSP90β was highly expressed in infiltrating tumor-associated macrophages in metastatic oral cancer (49).

Interestingly, Li and colleagues have reported that cytosolic HSP90 together with its co-chaperone CDC37 are important for the regulation of necroptosis (50). Mechanistically, receptor-interacting protein kinase 3 (RIP3) binds to HSP90-CDC37 while HSP90 inhibition disrupts RIP3 activation, thus blocking necroptosis (50).

HSP90α also interacts with B-cell lymphoma 2 (Bcl-2) –associated transcription factor 1 (Bclaf1) (51). Zhou and co-workers reported that HSP90 CTD domain inhibitor novobiocin resulted in proteasomal degradation of Bclaf, reduced *c-Myc* mRNA and inhibited hepatocellular carcinoma growth, suggesting that targeting HSP90 CTD domain may be a promising strategy for tumors with Bclaf upregulation (51). Cooper and colleagues showed that HSP90α/β also interacts with GSK3β/axin1/β-catenin (52). In another study, Wang and colleagues demonstrated that overexpression of HSP90β leads to growth, invasion and migration of gastric cancer cells (53). Mechanistically, HSP90β interacts with LRP5, leading to EMT, *via* activation of Akt and Wnt/β-catenin signaling pathways in gastric cancer cells (53). Taken together, HSP90α and HSP90β act through multiple signaling pathways, including c-Myc, Akt and Wnt/β-catenin (54).

Intriguingly, inactivation of ubiquitin-specific protease 22 (USP22), member of gene expression signature known as “death-from-cancer”, associates with lower HSP90β expression in mammary and colorectal cell lines (55). USP22-depleted tumor cells exhibited a high sensitivity to HSP90 inhibitor ganetespib, suggesting that targeting USP22 and HSP90β may prove effective for the treatment of breast and colorectal cancer (55). Recently, Pan and co-workers have shown that HSP90β stabilizes microtubule-associated serine/threonine kinase 1 (MAST1), a molecule associated with cisplatin resistance (56). Mechanistically, HSP90β binds to MAST1 and prevents its ubiquitination by CHIP and the ensuing degradation *via* proteasome (56). In this regard, HSP90 inhibitor 17-AAG has been shown to sensitize cells to cisplatin (56).

HSP90α and HSP90β also interact with HSP70 family members. Specifically, Moriya and co-workers demonstrated that HSP90α together with HSP70 ER member GRP78/BiP interact with PRDM14, a member of PR domain-containing family overexpressed in many tumors (57). In another study, Rozenberg and colleagues reported that HSP90β interacts with mortalin during complement activation (58). Results also showed that HSP90β competes with mortalin for binding to complement C9 (58). It appears that the interaction of HSP90β with mortalin protects tumor cells from complement-dependent cytotoxicity (CDC) (58).

Taking into account the important roles of HSP90α and HSP90β in tumor development, it is critical to identify HSP90 isoform-specific inhibitors. In this regard, Huck and colleagues demonstrated that protein-scaffold inhibitors preferentially bind HSP90α rather than HSP90β (59). In another study, Khandelwal and co-workers have designed a selective HSP90β inhibitor, which resulted in specific degradation of HSP90β clients (60). Collectively, HSP90α and HSP90β play a critical role in angiogenesis, invasion, metastasis, EMT and CDC, however, further studies are needed to identify the distinct functions of HSP90α and HSP90β in cancer development.

### HSP70/HSPA1A/1B and HSC70/HSPA8

HSP70 and the heat shock cognate protein 70 (HSC70) are stress-inducible and constitutive cytosolic isoforms encoded by *HSPA1A/1B* and *HSPA8*, respectively (61). HSP70 chaperone function involves co-chaperones, such as HOP, CHIP, HSP40, HSP70-interacting protein (Hip) and NEFs (13, 62–66). Co-chaperones assist HSP70 throughout its functional cycle in folding and degradation of its client proteins (13).

HSP70 is a multi-functional chaperone which has been implicated in various hallmarks of cancer [reviewed in (5)]. Mechanistically, HSP70 blocks apoptosis *via* inhibiting c-Jun N-terminal kinase (JNK), p38, apoptosis-inducing factor (AIF) and formation of death-inducing signaling complex (DISC) (67–70). Apart from apoptosis, HSP70 also regulates both necrosis by inhibiting JNK and autophagy by stabilizing lysosomal membranes (71–74). Furthermore, HSP70 is essential for survival of malignant cells as HSP70 protects tumor cells from oncogene-induced senescence program by regulating p53 and cyclin-dependent kinase Cdc2 (5, 72).

HSP70 also interacts with aminoacyl-transfer RNA synthetase-interacting multifunctional protein 2 (AIMP2) lacking exon 2 (AIMP-DX2) and HIF-1α, leading to angiogenesis, metastasis and tumor aggressiveness (75–78). Along this line, overexpression of HSP70 correlates with metastatic tumors (79). HSP70-peptide complexes isolated from hepatocellular carcinoma tissues promote EMT *via* p38 mitogen-activated protein kinase (MAPK) pathway (80). Additionally, HSP70 stabilizes E-cadherin/catenin complexes and Wiskott-Aldridge syndrome family member 3 (WASF3), thus regulating the metastatic process (81–84).

HSP70 plays critical role in tumor immunity. Several studies have shown that HSP70-peptide complexes induce cytotoxic T lymphocyte (CTL) response (85–87). In addition, Multhoff and colleagues reported that HSP70s on the tumor cells are recognized by NK cells (88). Moreover, HSP70-derived peptide TKD together with IL-2 or IL-15 can stimulate NK cells (89–91). This was further translated into a phase II clinical trial, where TKD peptide was used to pre-stimulate autologous NK cells for their adoptive transfer into patients with non-small cell lung carcinoma (92).

HSC70 is also involved in chaperone-assisted selective autophagy and endosomal microautophagy (eMI) (93–95). Li and colleagues reported that mitochondrial outer membrane protein FUNDC1 associates and delivers HSC70-peptide complex to mitochondria for its further ubiquitination by CHIP (96). HSC70 also interacts with Rab1A, a critical molecule for cancer cell survival (97). HSC70 inhibition downregulates Rab1A expression, while Rab1A inactivation leads to cell death *via* inhibition of autophagosome formation, suggesting that HSC70 promotes tumor survival by stabilizing Rab1A (97). HSC70-intreacting partners also include ASIC2, mutant forms of p53 and p73, proto-oncogenic form of Dbl and cell surface nucleolin (98–101).

Several studies reported that upon heat shock or oxidative stress HSC70 translocates from the cytoplasm into the nucleus (102, 103). Wang and colleagues reported that inhibition of nuclear HSC70 reduces cell growth upon heat shock (103). High expression of HSC70 has been observed in various tumors (104, 105). HSC70 was also identified as one of the proteins secreted by neuroblastoma cell lines in the conditioned media (106). Shan and colleagues illustrated that HSPA8 silencing dampens the cell proliferation and induces apoptosis in endometrial cancer cells (107). In another study, HSC70 depletion increased the expression of integrin β1, suggesting that HSC70 may promote invasion (108).

Mizukami and colleagues reported that fusion of HSC70 with CD4^+^T and CD8^+^ T cell epitopes elicited anti-tumor response (109). In another study, Zhang and colleagues demonstrated that fusion of HSC70-derived ATPase domain with tyrosinase-related protein 2 (TRP2) mounted CTL response in B16 melanoma, suggesting that HSC70-based immunotherapy approaches might prove effective for anti-cancer treatment (105, 110).

## Mitochondrial HSP90 and HSP70 in Cancer

### TRAP1

Tumor necrosis factor receptor-associated protein 1 (TRAP1) was initially discovered as a protein associated with the cytoplasmic domain of type 1 Tumor necrosis Factor Receptor-1 (TNFR1) (111, 112). The 75-kDa molecular chaperone, designated as HSP75, showed the ability to form complexes with the retinoblastoma protein (113). It then became clear that TRAP1 and HSP75 are identical molecules (112). TRAP1 functions as homodimer and requires ATP for its chaperone activity (114). TRAP1 has N-terminal mitochondrial targeting sequence that directs TRAP1 to mitochondrial matrix and is cleaved upon the import (115–117).

TRAP1 is highly expressed in mitochondria isolated from tumor cells compared to normal cells (3). Long lines of experimental evidence suggest that TRAP1 is involved in tumor metabolism and cytoprotection of cancer cells. Masuda and colleagues reported that induction of apoptosis by β-hydroxyisovalerylshikonin (β-HIVS) and topoisomerase II inhibitor VP16 in tumor cell lines is associated with the reduction in TRAP-1 expression (118). Moreover, inactivation of *TRAP1* by small interfering RNA (siRNA) in tumor cells treated with β-HIVS or VP16 induced the release of cytochrome *c*, pointing out an important role of TRAP1 in intrinsic apoptotic pathway (112, 118). In a subsequent study, Hua and colleagues demonstrated that granzyme M, a serine protease stored in granules of NK cells, acts on mitochondria and causes swelling, loss of transmembrane potential, production of reactive oxygen species (ROS) and cytochrome *c* release (119). Mechanistically, granzyme M cleaves TRAP1 leading to ROS accumulation and cell death (119). Kang and colleagues reported that TRAP1 and HSP90 in mitochondria interact with cyclophilin D and antagonize the mitochondrial permeability transition process (3, 112).

TRAP1 showed to be a critical regulator of mitochondrial metabolism. Sciacovelli and co-workers demonstrated that high expression of TRAP1 in tumor cells enhances neoplastic transformation (120). Specifically, TRAP1 forms complexes with succinate dehydrogenase (SDH) and inhibits its activity, contributing to Warburg phenotype (120). Warburg phenotype is characterized by preferential conversion of glucose to lactate, so that tumor cells mainly rely on glycolysis, an anaerobic metabolism for ATP production, even in the presence of oxygen (121). TRAP1 inhibits oxygen consumption rate and ATP synthesis by oxidative phosphorylation (OXPHOS) (120). Results also showed that TRAP1-expressing tumor cells have a high level of succinate, resulting in HIF-1α stabilization (120, 122). Along this line, Chae and co-workers reported that TRAP1 together with SDHB regulate HIF1α-dependent tumorigenesis (123). In another study, Yoshida and colleagues found that *TRAP1* knockout (KO) enhances mitochondrial respiration and suppresses glycolysis (124). Furthermore, *TRAP1* KO cells exhibited high levels of ATP, ROS production and cytochrome *c* oxidase (complex IV), a terminal enzyme in electron transport chain required for ATP production (124). Authors also showed that TRAP1 associates with c-Src and downregulates its activity (124). In addition, Park and colleagues demonstrated that interaction of TRAP1 with sirtuin-3 enhances mitochondrial respiration and reduces ROS production in glioma stem cells, thus supporting stemness (125).

Taking into account that full-length of TRAP1 is required for OXPHOS regulation, it has been suggested that TRAP1, similarly to HSP90, requires other chaperones for its OXPHOS function (117). In a recent study, Joshi and colleagues demonstrated that TRAP1 interacts with other mitochondrial chaperones, including HSPA9/GRP75, HSP60 and prohibitin as well as with OXPHOS-associated molecules, such as complex IV, complex II and ATP synthase (117). Interestingly, most of TRAP1 interactors, except for GRP75 and HSP60, had a preference for ATP-bound state (117).

Inactivation of TRAP1 showed to enhance invasion (124). Agliarulo and co-workers demonstrated that TRAP1 silencing promotes cell motility while simultaneously compromising the ability of cells to cope with stress, and this effect showed to be mediated *via* the AKT pathway (126). It is also interesting to point out that TRAP1 expression varies in different types of cancer. For example, low expression of TRAP1 correlated with high-grade cervical and bladder cancer, while high TRAP1 expression was found in colorectal carcinomas (124, 127). Therefore, further studies are required to understand the role of TRAP1 in mitochondrial bioenergetics, apoptotic mechanisms and its expression in specific types of cancer.

### GRP75/HSPA9/Mortalin/mtHSP70

Mortalin is found in mitochondria, ER, nucleus, cytosol, extracellular vesicles and on the cell surface (24, 128, 129). Mortalin shares 52% and 65% homology with stress-inducible isoform HSP70/HSPA1A and yeast mitochondrial HSP70 – SSC1, respectively (130). Similar to TRAP1, mortalin has a 46-amino acid mitochondrial targeting sequence that allows GRP75 to be localized in mitochondria (131). Mortalin is highly expressed in tumor tissues, leading to tumor growth, metastasis, angiogenesis and apoptosis resistance (132, 133). Ryu and co-workers used mutant mortalin, lacking the mitochondrial targeting sequence, to identify the presence of mortalin in the nucleus and, hence, they called it nuclear mortalin (129). Nuclear mortalin inhibits p53 and activates telomerase and heterogeneous nuclear ribonucleoprotein K (hnRNP-K) (129, 132, 134–136). Importantly, Lu and colleagues demonstrated that mortalin interacts with p53 in cancer cells under stress (136). Targeting mortalin-p53 interaction has resulted in p53-dependent apoptosis in tumor cells, suggesting that disruption of mortalin-p53 complex may be a promising strategy for anti-cancer therapy (136, 137).

Another strategy by which mortalin protects cancer cell from apoptosis involves HIF-1α (138). Recently, Mylonis and colleagues have reported that mortalin binds and mediates targeting of HIF-1α to the outer mitochondrial membrane, where HIF-1α blocks apoptosis when ERK is inactivated (138). HIF-1α release from the mitochondria under ERK inactivation resulted in induction of apoptosis (138).

Mortalin plays a critical role in epithelial-mesenchymal transition (EMT) (139). High expression of proteins involved in focal adhesion, PI3K-AKT and JAK-STAT signaling has been observed in mortalin - positive tumor cells (139). Furthermore, these cells exhibited high expression of mesenchymal markers, including vimentin, fibronectin, β-catenin and CK14, while the expression of epithelial markers (E-cadherin, CK8 and CK18) was reduced (139).

In a recent study, Yun and colleagues have reported that cells that overexpress mortalin had increased expression of cancer cell stemness markers, such as ABCG2, OCT-4, CD9, MRP1, ALDH1 and CD133 (132). Results had also shown that inactivation of mortalin by short hairpin RNA (shRNA) suppresses migration and invasion (132). Moreover, high expression of mortalin has correlated with resistance to therapies while mortalin silencing sensitized tumor cells to chemotherapeutic agents (132). In a recent study, Li and colleagues have demonstrated that NF-κB binds to mortalin promoter, leading to ovarian cancer cell proliferation (140). Conversely, NF-κB downregulation leads to reduction in mortalin expression (140).

Similar to TRAP1, mortalin plays an important role in mitochondrial bioenergetics (141). Mortalin is a major mitochondrial protein involved in mitochondrial import of proteins (142). Mortalin, bound to the translocase of the inner membrane-44 (TIM-44), imports the preprotein into the mitochondrial matrix, where mortalin refolds or transfers the preprotein to HSP60 chaperone (142–144). Inactivation of mortalin leads to a loss of mitochondrial membrane potential, reduction of oxygen consumption and induction of oxidative stress in medullary thyroid carcinoma (145).

## ER HSP90 and HSP70 in Cancer

### GRP94/HSP90B1/gp96/ERp99/Endoplasmin

The HSP90 member that resides in ER is GRP94 (146). GRP94 is targeted to ER by its N-terminal signal sequence that is cleaved upon GRP94 entry into the ER lumen where GRP94 resides due to its C-terminal KDEL sequence (146, 147). Another location where GRP94 has been identified is the cell surface (146). Several studies reported the presence of GRP94 on the surface of tumor cells and a small portion of immature thymocytes during early development, though the role of membrane-bound GRP94 is not yet clear (148, 149). Additionally, GRP94 functions as a dimer and unlike cytosolic HSP90s, has no known co-chaperones (146).

Unlike cytosolic HSP90 homologs, GRP94 is not upregulated in response to a high temperature, but rather is induced in response to ER stress, including glucose deprivation, hypoxia, B cell differentiation and perturbations of calcium or redox homeostasis (146, 150–154). Stress in ER machinery leads to cascades of signals known as unfolded protein response (UPR), which subsequently restores homeostasis or induces growth arrest and apoptosis (146, 155, 156).

Proper folding of proteins and quality control require collaboration between GRP94 and mitochondrial HSP70 family member GRP78 (146). Similar to HSP70-HSP90 collaboration, GRP78 binds to immunoglobulin (Ig) chains followed by GRP94 Ig folding in ER (146, 157, 158). Furthermore, GRP78-GRP94 forms ternary complex with client proteins in ER presumably for handling over the clients from GRP78 to GRP94 (146, 158).

GRP94 functions are not restricted to UPR, as GRP94 showed to be a critical immune chaperone [reviewed in (37)] (159). It has been shown that GRP94 is a chaperone for integrins and leucine-rich repeats domain 32 (LRRC32), also known as GARP, a docking protein for the membrane expression of transforming growth factor - β (TGF-β) (159, 160). Zhang and colleagues showed that GRP94 deletion in T regulatory cells leads to the loss of FOXP3, increased expression of interferon - γ (IFN-γ) and reduced bioavailability of TGF-β (160, 161). Since TGF-β plays critical roles in oncogenic processes, including EMT, angiogenesis, proliferation, metastasis and immune evasion, targeting GRP94 may prove effective for the development of anticancer therapies through the control of the expression of TGF-β (159).

Melendez and colleagues demonstrated GRP94 is expressed on the surface of breast cancer cells, whereas no expression of GRP94 was observed on the surface of non-malignant cells (162). Zheng and co-workers reported that GRP94 surface expression on tumor cells induces DC maturation and primes T cells, suggesting that GRP94 is a potent DC stimulator (163).

Besides immunologic functions, GRP94 regulates maturation of insulin-like growth factors (IGFs), which are essential prosurvival factors for tumor cells (159, 164). Hua and colleagues demonstrated that inactivation of GRP94 resulted in apoptosis of multiple myeloma cells *via* disruption of the Wnt-LRP6-survivin pathway (165). Results also showed that GRP94 inhibition blocked multiple myeloma growth in mouse xenograft model, suggesting that GRP94 may be a promising target for the treatment of multiple myeloma (165).

With the use of GRP94-selective inhibitor PU-WS13, Patel and colleagues demonstrated that GRP94 plays an important role in plasma membrane HER2 stability, and inactivation of GRP94 resulted in reduction of HER2-overexpressing tumor cell viability (166). Mechanistically, inhibition of GRP94 leads to the translocation of HER2 to early endosomes and plasma-membrane adjacent lysosomes (166). Along this line, membrane GRP94 interacts with HER2 and facilitates its dimerization, contributing to cell proliferation (167). Targeting GRP94 with a monoclonal antibody reduced growth and increased apoptosis in breast cancer cells (167). In another study, targeting GRP94 with the W9 monoclonal antibody sensitized BRAF^V600E^ melanoma cells to BRAF inhibitors (168). Taken together, GRP94 plays crucial role in UPR, tumor immunity and promotes cancer *via* its client network. GRP94-based immunotherapy approaches represent promising strategies for anti-cancer therapy, however, this requires further investigation.

### GRP78/HSPA5/BiP

GRP78 performs various cellular functions, including folding, degradation, transport of peptides across ER membrane and regulation of calcium homeostasis (169, 170). Similar to cytosolic HSP70 homologs, GRP78 is composed of N-terminal ATPase domain and C-terminal substrate-binding domain (SBD) (168). Due to its ER retention motif, GRP78 primarily resides in ER, but it has also been observed in mitochondria, cytoplasm, cell surface, nucleus and extracellular vesicles (8, 171, 172). Similar to GRP94, GRP78 chaperone plays critical role in UPR, initiated upon ER stress (171). GRP78 inactivation results in spontaneous activation of UPR, expansion of ER lumen and induction of GRP94 expression (173).

Another process that is activated upon ER stress and involves GRP78 is autophagy. High expression of GRP78 increased autophagosome formation in estrogen receptor-positive breast cancer cells (174, 175). Mechanistically, elevated expression of GRP78 activates AMP-activated protein kinase (AMPK) and tuberous sclerosis 2 (TSC2), both of which inhibit mechanistic target of rapamycin (mTOR), resulting in initiation of autophagy (174, 175). Silencing of GRP78 leads to inhibition of autophagosome formation (173). Furthermore, Li and colleagues demonstrated that high expression of GRP78 activates the Class III phosphatidylinositol 3-kinase (PI3K)-mediated autophagy pathway and induces degradation of IKKβ, leading to inhibition of the NF-κB pathway, at the same time altering expression of pyruvate kinase M2 and HIF-1α (176). Along this line, under stress conditions, GRP78 binds to cytosolic misfolded proteins and SQSTM1/p62 (171, 177, 178). Interaction with p62 leads to SQSTM1/p62 conformational change, favoring cargo delivery into autophagosome for its further degradation into amino acids (171, 177, 178). Malek and co-workers reported that treatment with the proteasome inhibitor bortezomib induces GRP78 and GRP78-mediated autophagy in myeloma cells (179). Inhibition of GRP78 followed by bortezomib treatment disrupted autophagy and enhanced anti-tumor effect (179). In a recent study, Wu and colleagues have demonstrated that the GRP78 inhibitor HA15 promoted apoptosis which was accompanied with UPR and autophagy in lung cancer cells (180).

ER stress and UPR induce GRP78, resulting in its translocation to mitochondrial compartments, including intermembrane space, inner membrane and matrix (181). Hayashi and colleagues demonstrated that GRP78 forms complex with sigma-1 receptor (Sig-1R), ER calcium-sensitive co-chaperone in mitochondrion-associated membrane (182). Under ER stress, Sig-1R dissociates from GRP78 and binds to inositol 1,4,5- trisphosphate receptors, promoting a prolonged calcium influx from ER into mitochondria (182).

Recently, Ni and co-workers have identified a novel cytosolic GRP78 isoform (GRP78va) generated by alternative splicing (183). Results showed that GRP78va is upregulated in human leukemia cell lines, as well as in primary leukemia cells obtained from patients (183). GRP78va lacks ER retention signaling peptide and specifically activates ER kinase PERK (183). Mechanistically, GRP78va interacts with P58IPK, an inhibitor of PERK, and antagonizes its inhibitory activity (183). Inactivation of GRP78va decreased survival, whereas overexpression promoted survival of leukemia cells, suggesting that high expression of the cytosolic GRP78 isoform protects cancer cells from cell death (183). GRP78 may also translocate to cytosol through the ER-associated degradation (ERAD) pathway and *via* Bax/Bak-dependent changes, affecting ER permeability upon ER stress-induced apoptosis (184, 185).

GRP78 has also been detected in the nucleus (186–188). Matsumoto and colleagues used gilvocarcin V (GV), anti-tumor antibiotic that promotes protein-DNA cross-linking when photoactivated by near UV-light, to show that GRP78 lacking hydrophobic leader sequence was selectively cross-linked to DNA in human fibroblasts (189). In another study, Zhai and co-workers demonstrated that inactivation of GRP78 sensitizes cells to UVC-induced cell death, suggesting a protective role of GRP78 against DNA damage (186, 190).

High expression of GRP78 was observed in various types of cancer such as colon, lung, prostate, myeloma, leukemia and breast cancer and showed to correlate with unfavorable clinical outcome (179, 191, 192). Biallelic inactivation of both *PTEN* and *GRP78* inhibited AKT activation and tumorigenesis in prostate epithelium (193). This was further supported by the finding that antibody directed against COOH-terminal domain of GRP78 inhibited growth and AKT activity in prostate cancer cell lines (194). In another study, Cook and colleagues demonstrated that GRP78 inactivation inhibits *de novo* fatty acid synthesis in breast cancer cells (195). Combination of tamoxifen and GRP78-targeting morpholino antisense oligonucleotides resulted in increased ROS production and cell death (195). Intriguingly, GRP78 inactivation downregulated the expression of innate immune checkpoint CD47 in breast cancer cells, whereas reduction of GRP78 in normal mammary tissue increased the expression of CD47 and macrophage infiltration (195). Recently, the same research team has demonstrated that co-expression of CD47 and GRP78 associated with a poor outcome in breast cancer patients (196).

Induction of UPR affects sensitivity of cells to chemotherapeutic agents (197). In this regard, Reddy and co-workers demonstrated that elevated GRP78 expression inhibits apoptosis in cells treated with topoisomerase inhibitors (187). Mechanistically, etoposide treatment leads to the activation of caspase-7, while elevated expression of GRP78 inhibits caspase-7 activation (187). Along this line, several studies showed that GRP78 forms a complex with caspase-7 and caspase -12 and prevents release of caspase-12 from ER, suggesting that one of the mechanisms by which GRP78 blocks cell death is by inhibiting caspase activation (187, 198). In another study, Lee and colleagues observed elevated expression of GRP78 in 5-fluorouracil (5-FU)-resistant colorectal cancer cells (180). GRP78 inhibition in cells treated with 5-FU led to apoptosis through the activation of caspase-3 (180). Furthermore, GRP78 promoted cell survival *via* the activation of PI3K-AKT-mTOR signaling pathway (180).

Recently, Dauer and colleagues have demonstrated that GRP78 silencing leads to a slower proliferation rate, reduction in colony formation and downregulation of genes involved in self-renewal in pancreatic cancer cells (199). Furthermore, GRP78 silencing affected the redox balance leading to lipid-peroxidation and higher ROS production (199). Chang and co-workers reported that overexpression of GRP78/p-PERK signaling pathway activates nuclear factor-erythroid 2-related factor (NRF2), leading to enhanced expression of glycolytic enzymes and stemness markers in head and neck squamous cell carcinoma, thus, supporting Warburg phenotype and cancer cell stemness (200). Taken together, GRP78s may change its location and mediate various processes, including UPR, Warburg phenotype, stemness, apoptosis, autophagy and innate immune responses.

## Discussion

**Figure 2:**
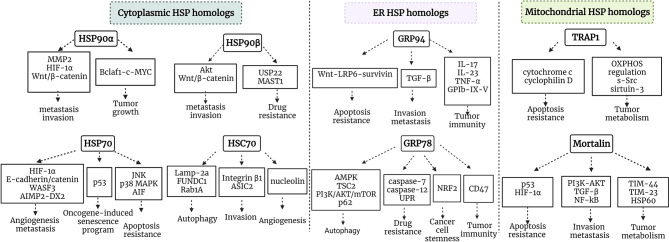
Schematic representation of HSP90/HSP70 signaling pathways in cancer. HSP90α and HSP90β are cytosolic stress-inducible and constitutive members of HSP90 family, respectively, which support tumor development *via* multiple signaling pathways. HSP70 and HSC70 are two main cytosolic stress-inducible and constitutive members of HSP70 family, respectively, which play critical roles in the regulation of apoptosis, autophagy, oncogene-induced senescence program, angiogenesis, invasion and metastasis. GRP94 and GRP78 are ER HSP90 and HSP70 members, which play an important role in the regulation of apoptosis, invasion, metastasis, autophagy, drug resistance, cancer cell stemness and tumor immunity. TRAP1 is a mitochondrial HSP90, which plays critical role in tumor metabolism and cytoprotection of cancer cells. Mortalin is a mitochondrial HSP70 family member playing an important role in tumor metabolism, regulation of apoptosis, invasion and metastasis. MMP9, matrix metalloproteinase 9; OXPHOS, oxidative phosphorylation; Bclaf1, B-cell lymphoma 2 -associated transcription factor 1; HIF-1, hypoxia-inducible factor 1; MAST1, microtubule-associated serine/threonine kinase 1; TGF-β, transforming growth factor β; PI3K, phosphatidylinositol 3-kinase; WASF3, Wiskott-Aldridge syndrome family member 3; TIM-44, translocase of inner membrane-44; NRF2, nuclear factor-erythroid 2-related factor; AIMP-DX2, aminoacyl-transfer RNA synthetase-interacting multifunctional protein 2 (AIMP2) lacking exon 2; MAPK, mitogen-activated protein kinase; TSC2, tuberous sclerosis 2; mTOR, mechanistic target of rapamycin.

Residing in various cellular compartments, HSP70 and HSP90 isoforms perform distinct functions within a cancer cell. HSP70 and HSP90 homologs are critical regulators of UPR, mitochondrial bioenergetics, lipid metabolism, apoptosis, innate and adaptive immune responses. Apart from their primary locations, HSP70 and HSP90 isoforms may translocate and accumulate in specific locations inside the cell under various stress conditions, thus supporting tumorigenesis. Furthermore, HSP homologs may be released into extracellular space and acquire different functions. HSP90 and HSP70 cytosolic, ER and mitochondrial isoforms support tumor growth and development *via* different signaling pathways (**Figure 2**). Concurrently, different HSP homologs may also act through the same mechanism. For example, inhibiting the interaction between HSP90α and Bclaf1 leads to the downregulation of mature *c-Myc* mRNA, while *Myc* silencing decreases *TRAP1* mRNA (51, 201). Furthermore, Zavareh and colleagues demonstrated that HSP90 inhibition downregulates the expression of immune checkpoint PD-L1 on the surface of tumor cells *via* the regulation of c-Myc (202). HSP105 inhibition also downregulates c-Myc (203). Therefore, targeting specific molecular pathways by inhibiting HSP homologs may be effective against tumors with the dysregulation of specific signaling pathways, however, it should be taken into account that blocking a specific HSP isoform may have an effect on other HSP homologs, and this requires further investigation.

Even though considerable progress has been made in assessing intracellular and extracellular functions of HSP70 and HSP90 in cancer, a lot is still unclear. For example, the effect of various HSP70 and HSP90-based therapies on the distribution of HSP70 and HSP90 homologs across cellular compartments and their release in extracellular space is unknown and requires further investigation. It is also important to differentiate between two HSP90 cytosolic isoforms and assess their individual functions in cancer. Furthermore, since HSP90 and HSP70 play critical roles in innate and adaptive immune responses, it is important to understand intracellular HSP70 and HSP90 immune functions in cancer. Elucidating intracellular and extracellular roles of individual HSP70 and HSP90 homologs may provide further clues on the release of HSP70 and HSP90 in the tumor microenvironment and help in the development of more effective HSP70 and HSP90-based therapies.

## Conclusion

HSP90 and HSP70 are two powerful chaperone machineries involved in almost all stages of tumor development. HSP90 and HSP70 homologs are implicated in the regulation of apoptosis, UPR, lipid metabolism, metastasis, angiogenesis, autophagy, innate and adaptive immune responses, acting *via* different signaling pathways. Further understanding of molecular mechanisms of specific HSP90 and HSP70 homologs inside and outside the cell may provide clues for the discovery of novel anti-cancer therapies.

## Author Contributions

ZA wrote and revised the manuscript. AA contributed to editing and revision of the manuscript. YM and LG provided an administration support. All authors contributed to the article and approved the submitted version.

## Funding

This research was funded by RFBR, project number 20-315-90081.

## Conflict of Interest

The authors declare that the research was conducted in the absence of any commercial or financial relationships that could be construed as a potential conflict of interest.

## Publisher’s Note

All claims expressed in this article are solely those of the authors and do not necessarily represent those of their affiliated organizations, or those of the publisher, the editors and the reviewers. Any product that may be evaluated in this article, or claim that may be made by its manufacturer, is not guaranteed or endorsed by the publisher.

## References

[1] Young JC Moarefi I Hartl FU. Hsp90: A Specialized But Essential Protein-Folding Tool. J Cell Biol (2001) 154(2):267–73. doi: 10.1083/jcb.200104079 PMC2150759 11470816

[2] Young JC Hoogenraad NJ Hartl FU. Molecular Chaperones Hsp90 and Hsp70 Deliver Preproteins to the Mitochondrial Import Receptor Tom70. Cell (2003) 112(1):41–50. doi: 10.1016/S0092-8674(02)01250-3 12526792

[3] Kang BH Plescia J Dohi T Rosa J Doxsey SJ Altieri DC. Regulation of Tumor Cell Mitochondrial Homeostasis by an Organelle-Specific Hsp90 Chaperone Network. Cell (2007) 131(2):257–70. doi: 10.1016/j.cell.2007.08.028 17956728

[4] Pearl LH Prodromou C. Structure and Mechanism of the Hsp90 Molecular Chaperone Machinery. Annu Rev Biochem (2006) 75(1):271–94. doi: 10.1146/annurev.biochem.75.103004.142738 16756493

[5] Albakova Z Armeev GA Kanevskiy LM Kovalenko EI Sapozhnikov AM. HSP70 Multi-Functionality in Cancer. Cells (2020) 9(3):1–26. doi: 10.3390/cells9030587 PMC7140411 32121660

[6] Miyata Y Nakamoto H Neckers L. The Therapeutic Target Hsp90 and Cancer Hallmarks. Curr Pharm Des (2013) 19(3):347–65. doi: 10.2174/138161213804143725 PMC7553218 22920906

[7] Albakova Z Mangasarova Y Sapozhnikov A. Heat Shock Proteins in Lymphoma Immunotherapy. Front Immunol (2021) 12(769). doi: 10.3389/fimmu.2021.660085 PMC8012763 33815422

[8] Albakova Z Siam MKS Sacitharan PK Ziganshin RH Ryazantsev DY Sapozhnikov AM. Extracellular Heat Shock Proteins and Cancer: New Perspectives. Trans Oncol (2021) 14(2):100995. doi: 10.1016/j.tranon.2020.100995 PMC7749402 33338880

[9] Johnson JL. Evolution and Function of Diverse Hsp90 Homologs and Cochaperone Proteins. Biochim Biophys Acta (BBA) - Mol Cell Res (2012) 1823(3):607–13. doi: 10.1016/j.bbamcr.2011.09.020 22008467

[10] Kampinga HH Hageman J Vos MJ Kubota H Tanguay RM Bruford EA. Guidelines for the Nomenclature of the Human Heat Shock Proteins. Cell Stress Chaperones (2009) 14(1):105–11. doi: 10.1007/s12192-008-0068-7 PMC2673902 18663603

[11] Southworth DR Agard DA. Species-Dependent Ensembles of Conserved Conformational States Define the Hsp90 Chaperone Atpase Cycle. Mol Cell (2008) 32(5):631–40. doi: 10.1016/j.molcel.2008.10.024 PMC2633443 19061638

[12] Lavery LA Partridge JR Ramelot TA Elnatan D Kennedy MA Agard DA. Structural Asymmetry in the Closed State of Mitochondrial Hsp90 (TRAP1) Supports a Two-Step ATP Hydrolysis Mechanism. Mol Cell (2014) 53(2):330–43. doi: 10.1016/j.molcel.2013.12.023 PMC3947485 24462206

[13] Rosenzweig R Nillegoda NB Mayer MP Bukau B. The Hsp70 Chaperone Network. Nat Rev Mol Cell Biol (2019) 20(11):665–80. doi: 10.1038/s41580-019-0133-3 31253954

[14] Schopf FH Biebl MM Buchner J. The HSP90 Chaperone Machinery. Nat Rev Mol Cell Biol (2017) 18(6):345–60. doi: 10.1038/nrm.2017.20 28429788

[15] Jackson SE. Hsp90: Structure and Function, in Molecular Chaperones. Jackson S, editor. Berlin, Heidelberg: Springer Berlin Heidelberg (2013) p. 155–240. 10.1007/128_2012_356 22955504

[16] Taipale M Jarosz DF Lindquist S. HSP90 at the Hub of Protein Homeostasis: Emerging Mechanistic Insights. Nat Rev Mol Cell Biol (2010) 11(7):515–28. doi: 10.1038/nrm2918 20531426

[17] Wang RY-R Noddings CM Kirschke E Myasnikov AG Johnson JL Agard DA. Structure of Hsp90–Hsp70–Hop–GR Reveals the Hsp90 Client-Loading Mechanism. Nature (2021), 1–27. doi: 10.1038/s41586-021-04252-1 PMC9179170 34937942

[18] Wayne N Bolon DN. Dimerization of Hsp90 is Required for In Vivo Function. J Biol Chem (2007) 282(48):35386–95. doi: 10.1074/jbc.M703844200 17908693

[19] Stankiewicz M Nikolay R Rybin V Mayer MP. CHIP Participates in Protein Triage Decisions by Preferentially Ubiquitinating Hsp70-Bound Substrates. FEBS J (2010) 277(16):3353–67. doi: 10.1111/j.1742-4658.2010.07737.x 20618441

[20] Jacobs AT Marnett LJ. Systems Analysis of Protein Modification and Cellular Responses Induced by Electrophile Stress. Accounts Chem Res (2010) 43(5):673–83. doi: 10.1021/ar900286y PMC2873822 20218676

[21] Wawrzynow B Zylicz A Zylicz M. Chaperoning the Guardian of the Genome. The Two-Faced Role of Molecular Chaperones in P53 Tumor Suppressor Action. Biochim Biophys Acta (BBA) - Rev Cancer (2018) 1869(2):161–74. doi: 10.1016/j.bbcan.2017.12.004 29355591

[22] Dahiya V Agam G Lawatscheck J Rutz DA Lamb DC Buchner J. Coordinated Conformational Processing of the Tumor Suppressor Protein P53 by the Hsp70 and Hsp90 Chaperone Machineries. Mol Cell (2019) 74(4):816–830.e7. doi: 10.1016/j.molcel.2019.03.026 31027879

[23] Boysen M Kityk R Mayer MP. Hsp70- and Hsp90-Mediated Regulation of the Conformation of P53 DNA Binding Domain and P53 Cancer Variants. Mol Cell (2019) 74(4):831–843.e4. doi: 10.1016/j.molcel.2019.03.032 31027880

[24] Shin BK Wang H Yim AM Le Naour F Brichory F Jang JH. Global Profiling of the Cell Surface Proteome of Cancer Cells Uncovers an Abundance of Proteins With Chaperone Function. J Biol Chem (2003) 278(9):7607–16. doi: 10.1074/jbc.M210455200 12493773

[25] Cid C Regidor I Poveda PD Alcazar A. Expression of Heat Shock Protein 90 at the Cell Surface in Human Neuroblastoma Cells. Cell Stress Chaperones (2009) 14(3):321–7. doi: 10.1007/s12192-008-0076-7 PMC2728257 18800240

[26] Sidera K Gaitanou M Stellas D Matsas R Patsavoudi E. A Critical Role for HSP90 in Cancer Cell Invasion Involves Interaction With the Extracellular Domain of HER-2. J Biol Chem (2008) 283(4):2031–41. doi: 10.1074/jbc.M701803200 18056992

[27] Crescitelli R Lässer C Jang SC Cvjetkovic A Malmhäll C Karimi N. Subpopulations of Extracellular Vesicles From Human Metastatic Melanoma Tissue Identified by Quantitative Proteomics After Optimized Isolation. J Extracell Vesicles (2020) 9(1):1722433–1722433. doi: 10.1080/20013078.2020.1722433 32128073 PMC7034452

[28] Dhondt B Geeurickx E Tulkens J Van Deun J Vergauwen G Lippens L. Unravelling the Proteomic Landscape of Extracellular Vesicles in Prostate Cancer by Density-Based Fractionation of Urine. J Extracell Vesicles (2020) 9(1):1736935. doi: 10.1080/20013078.2020.1736935 32284825 PMC7144211

[29] Chen Y Xie Y Xu L Zhan S Xiao Y Gao Y. Protein Content and Functional Characteristics of Serum-Purified Exosomes From Patients With Colorectal Cancer Revealed by Quantitative Proteomics. Int J Cancer (2017) 140(4):900–13. doi: 10.1002/ijc.30496 27813080

[30] An M Lohse I Tan Z Zhu J Wu J Kurapati H. Quantitative Proteomic Analysis of Serum Exosomes From Patients With Locally Advanced Pancreatic Cancer Undergoing Chemoradiotherapy. J Proteome Res (2017) 16(4):1763–72. doi: 10.1021/acs.jproteome.7b00024 PMC5462613 28240915

[31] García-Silva S Benito-Martín A Sánchez-Redondo S Hernández-Barranco A Ximénez-Embún P Nogués L. Use of Extracellular Vesicles From Lymphatic Drainage as Surrogate Markers of Melanoma Progression and BRAF (V600E) Mutation. J Exp Med (2019) 216(5):1061–70. doi: 10.1084/jem.20181522 PMC6504207 30975894

[32] Federici C Shahaj E Cecchetti S Camerini S Casella M Iessi E. Natural-Killer-Derived Extracellular Vesicles: Immune Sensors and Interactors. Front Immunol (2020) 11:262–2. doi: 10.3389/fimmu.2020.00262 PMC7082405 32231660

[33] Perez-Hernandez D Gutiérrez-Vázquez C Jorge I López-Martín S Ursa A Sánchez-Madrid F. The Intracellular Interactome of Tetraspanin-Enriched Microdomains Reveals Their Function as Sorting Machineries Toward Exosomes. J Biol Chem (2013) 288(17):11649–61. doi: 10.1074/jbc.M112.445304 PMC3636856 23463506

[34] Kowal J Arras G Colombo M Jouve M Morath JP Primdal-Bengtson B. Proteomic Comparison Defines Novel Markers to Characterize Heterogeneous Populations of Extracellular Vesicle Subtypes. Proc Natl Acad Sci USA (2016) 113(8):E968–77. doi: 10.1073/pnas.1521230113 PMC4776515 26858453

[35] Garcia BA Smalley DM Cho, Shabanowitz J Ley K Hunt DF. The Platelet Microparticle Proteome. J Proteome Res (2005) 4(5):1516–21. doi: 10.1021/pr0500760 16212402

[36] Dalli J Montero-Melendez T Norling LV Yin X Hinds C Haskard D. Heterogeneity in Neutrophil Microparticles Reveals Distinct Proteome and Functional Properties. Mol Cell Proteomics (2013) 12(8):2205–19. doi: 10.1074/mcp.M113.028589 PMC3734580 23660474

[37] Albakova Z Mangasarova Y. The HSP Immune Network in Cancer. Front Immunol (2021) 12(5162). doi: 10.3389/fimmu.2021.796493 PMC8669653 34917098

[38] Albakova Z Norinho DD Mangasarova Y Sapozhnikov A. Heat Shock Proteins in Urine as Cancer Biomarkers. Front Med (2021) 8(1748). doi: 10.3389/fmed.2021.743476 PMC8531591 34692733

[39] Mittal S Rajala MS. Heat Shock Proteins as Biomarkers of Lung Cancer. Cancer Biol Ther (2020) 21(6):477–85. doi: 10.1080/15384047.2020.1736482 PMC7515496 32228356

[40] Caruso Bavisotto C Cappello F Macario AJL Conway de Macario E Logozzi M Fais S. Exosomal HSP60: A Potentially Useful Biomarker for Diagnosis, Assessing Prognosis, and Monitoring Response to Treatment. Expert Rev Mol Diagnostics (2017) 17(9):815–22. doi: 10.1080/14737159.2017.1356230 28718351

[41] Balázs M Zsolt H László G Gabriella G Lilla T Gyula O. Serum Heat Shock Protein 70, as a Potential Biomarker for Small Cell Lung Cancer. Pathol Oncol Res (2017) 23(2):377–83. doi: 10.1007/s12253-016-0118-x 27704355

[42] Dutta SK Girotra M Singla M Dutta A Otis Stephen F Nair PP. Serum HSP70: A Novel Biomarker for Early Detection of Pancreatic Cancer. Pancreas (2012) 41(4):530–4. doi: 10.1097/MPA.0b013e3182374ace PMC4193547 22158074

[43] Chanteloup G Cordonnier M Isambert N Bertaut A Hervieu A Hennequin A. Monitoring HSP70 Exosomes in Cancer Patients’ Follow Up: A Clinical Prospective Pilot Study. J Extracell Vesicles (2020) 9(1):1766192–1766192. doi: 10.1080/20013078.2020.1766192 32595915 PMC7301715

[44] Zuehlke AD Beebe K Neckers L Prince T. Regulation and Function of the Human HSP90AA1 Gene. Gene (2015) 570(1):8–16. doi: 10.1016/j.gene.2015.06.018 26071189 PMC4519370

[45] Prince TL Kijima T Tatokoro M Lee S Tsutsumi S Yim K. Client Proteins and Small Molecule Inhibitors Display Distinct Binding Preferences for Constitutive and Stress-Induced HSP90 Isoforms and Their Conformationally Restricted Mutants. PloS One (2015) 10(10):e0141786. doi: 10.1371/journal.pone.0141786 26517842 PMC4627809

[46] Taipale M Krykbaeva I Koeva M Kayatekin C Westover KD Karras GI. Quantitative Analysis of HSP90-Client Interactions Reveals Principles of Substrate Recognition. Cell (2012) 150(5):987–1001. doi: 10.1016/j.cell.2012.06.047 22939624 PMC3894786

[47] Dong H Zou M Bhatia A Jayaprakash P Hofman F Ying Q. Breast Cancer MDA-MB-231 Cells Use Secreted Heat Shock Protein-90alpha (Hsp90α) to Survive a Hostile Hypoxic Environment. Sci Rep (2016) 6:20605–5. doi: 10.1038/srep20605 PMC4742873 26846992

[48] Sahu D Zhao Z Tsen F Cheng C-F Park R Situ AJ. A Potentially Common Peptide Target in Secreted Heat Shock Protein-90α for Hypoxia-Inducible Factor-1α-Positive Tumors. Mol Biol Cell (2012) 23(4):602–13. doi: 10.1091/mbc.e11-06-0575 PMC3279389 22190738

[49] Ono K Sogawa C Kawai H Tran MT Taha EA Lu Y. Triple Knockdown of CDC37, HSP90-Alpha and HSP90-Beta Diminishes Extracellular Vesicles-Driven Malignancy Events and Macrophage M2 Polarization in Oral Cancer. J Extracell Vesicles (2020) 9(1):1769373–1769373. doi: 10.1080/20013078.2020.1769373 33144925 PMC7580842

[50] Li D Xu T Cao Y Wang H Li L Chen S. A Cytosolic Heat Shock Protein 90 and Cochaperone CDC37 Complex is Required for RIP3 Activation During Necroptosis. Proc Natl Acad Sci USA (2015) 112(16):5017–22. doi: 10.1073/pnas.1505244112 PMC4413296 25852146

[51] Zhou X Wen Y Tian Y He M Ke X Huang Z. Heat Shock Protein 90α-Dependent B-Cell-2-Associated Transcription Factor 1 Promotes Hepatocellular Carcinoma Proliferation by Regulating MYC Proto-Oncogene C-MYC Mrna Stability. Hepatol (Baltimore Md.) (2019) 69(4):1564–81. doi: 10.1002/hep.30172 PMC6586158 30015413

[52] Cooper LC Prinsloo E Edkins AL Blatch GL. Hsp90α/β Associates With the GSK3β/Axin1/Phospho-β-Catenin Complex in the Human MCF-7 Epithelial Breast Cancer Model. Biochem Biophys Res Commun (2011) 413(4):550–4. doi: 10.1016/j.bbrc.2011.08.136 21925151

[53] Wang H Deng G Ai M Xu Z Mou T Yu J. Hsp90ab1 Stabilizes LRP5 to Promote Epithelial-Mesenchymal Transition via Activating of AKT and Wnt/β-Catenin Signaling Pathways in Gastric Cancer Progression. Oncogene (2019) 38(9):1489–507. doi: 10.1038/s41388-018-0532-5 PMC6372478 30305727

[54] Lochhead PA Kinstrie R Sibbet G Rawjee T Morrice N Cleghon V. A Chaperone-Dependent Gsk3β; Transitional Intermediate Mediates Activation-Loop Autophosphorylation. Mol Cell (2006) 24(4):627–33. doi: 10.1016/j.molcel.2006.10.009 17188038

[55] Kosinsky RL Helms M Zerche M Wohn L Dyas A Prokakis E. USP22-Dependent HSP90AB1 Expression Promotes Resistance to HSP90 Inhibition in Mammary and Colorectal Cancer. Cell Death Dis (2019) 10(12):911–1. doi: 10.1038/s41419-019-2141-9 PMC6892875 31801945

[56] Pan C Chun J Li D Boese AC Li J Kang J. Hsp90B Enhances MAST1-Mediated Cisplatin Resistance by Protecting MAST1 From Proteosomal Degradation. J Clin Invest (2019) 129(10):4110–23. doi: 10.1172/JCI125963 PMC6763259 31449053

[57] Moriya C Taniguchi H Nagatoishi S Igarashi H Tsumoto K Imai K. PRDM14 Directly Interacts With Heat Shock Proteins HSP90α and Glucose-Regulated Protein 78. Cancer Sci (2018) 109(2):373–83. doi: 10.1111/cas.13458 PMC5797828 29178343

[58] Rozenberg P Ziporen L Gancz D Saar-Ray M Fishelson Z. Cooperation Between Hsp90 and Mortalin/GRP75 in Resistance to Cell Death Induced by Complement C5b-9. Cell Death Dis (2018) 9(2):150. doi: 10.1038/s41419-017-0240-z 29396434 PMC5833442

[59] Huck JD Que NLS Sharma S Taldone T Chiosis G Gewirth DT. Structures of Hsp90α and Hsp90β Bound to a Purine-Scaffold Inhibitor Reveal an Exploitable Residue for Drug Selectivity. Proteins (2019) 87(10):869–77. doi: 10.1002/prot.25750 PMC6718336 31141217

[60] Khandelwal A Kent CN Balch M Peng S Mishra SJ Deng J. Structure-Guided Design of an Hsp90β N-Terminal Isoform-Selective Inhibitor. Nat Commun (2018) 9(1):425–5. doi: 10.1038/s41467-017-02013-1 PMC5789826 29382832

[61] Lindquist S Craig EA. The Heat-Shock Proteins. Annu Rev Genet (1988) 22(1):631–77. doi: 10.1146/annurev.ge.22.120188.003215 2853609

[62] Laufen T Mayer MP Beisel C Klostermeier D Mogk A Reinstein J. Mechanism of Regulation of Hsp70 Chaperones by Dnaj Cochaperones. Proc Natl Acad Sci (1999) 96(10):5452. doi: 10.1073/pnas.96.10.5452 10318904 PMC21880

[63] Mayer MP Bukau B. Hsp70 Chaperones: Cellular Functions and Molecular Mechanism. Cell Mol Life Sci (2005) 62(6):670–84. doi: 10.1007/s00018-004-4464-6 PMC2773841 15770419

[64] Li Z Hartl FU Bracher A. Structure and Function of Hip, an Attenuator of the Hsp70 Chaperone Cycle. Nat Struct Mol Biol (2013) 20(8):929–35. doi: 10.1038/nsmb.2608 23812373

[65] Bracher A Verghese J. The Nucleotide Exchange Factors of Hsp70 Molecular Chaperones. Front Mol Biosci (2015) 2:10–0. doi: 10.3389/fmolb.2015.00010 PMC4753570 26913285

[66] Ballinger CA Connell P Wu Y Hu Z Thompson LJ Yin LY. Identification of CHIP, a Novel Tetratricopeptide Repeat-Containing Protein That Interacts With Heat Shock Proteins and Negatively Regulates Chaperone Functions. Mol Cell Biol (1999) 19(6):4535–45. doi: 10.1128/MCB.19.6.4535 PMC104411 10330192

[67] Gao Y Han C Huang H Xin Y Xu Y Luo L. Heat Shock Protein 70 Together With its Co-Chaperone CHIP Inhibits TNF-Alpha Induced Apoptosis by Promoting Proteasomal Degradation of Apoptosis Signal-Regulating Kinase1. Apoptosis (2010) 15(7):822–33. doi: 10.1007/s10495-010-0495-7 20349136

[68] Ravagnan L Gurbuxani S Susin SA Maisse C Daugas E Zamzami N. Heat-Shock Protein 70 Antagonizes Apoptosis-Inducing Factor. Nat Cell Biol (2001) 3(9):839–43. doi: 10.1038/ncb0901-839 11533664

[69] Guo F Sigua C Bali P George P Fiskus W Scuto A. Mechanistic Role of Heat Shock Protein 70 in Bcr-Abl-Mediated Resistance to Apoptosis in Human Acute Leukemia Cells. Blood (2005) 105(3):1246–55. doi: 10.1182/blood-2004-05-2041 15388581

[70] Gabai VL Mabuchi K Mosser DD Sherman MY. Hsp72 and Stress Kinase C-Jun N-Terminal Kinase Regulate the Bid-Dependent Pathway in Tumor Necrosis Factor-Induced Apoptosis. Mol Cell Biol (2002) 22(10):3415–24. doi: 10.1128/MCB.22.10.3415-3424.2002 PMC133785 11971973

[71] Nylandsted J Gyrd-Hansen M Danielewicz A Fehrenbacher N Lademann U Høyer-Hansen M. Heat Shock Protein 70 Promotes Cell Survival by Inhibiting Lysosomal Membrane Permeabilization. J Exp Med (2004) 200(4):425–35. doi: 10.1084/jem.20040531 PMC2211935 15314073

[72] Yaglom JA Ekhterae D Gabai VL Sherman MY. Regulation of Necrosis of H9c2 Myogenic Cells Upon Transient Energy Deprivation. Rapid Deenergization of Mitochondria Precedes Necrosis and is Controlled by Reactive Oxygen Species, Stress Kinase JNK, HSP72 and ARC. J Biol Chem (2003) 278(50):50483–96. doi: 10.1074/jbc.M306903200 14523009

[73] Daugaard M Kirkegaard-Sørensen T Ostenfeld MS Aaboe M Høyer-Hansen M Ørntoft TF. Lens Epithelium-Derived Growth Factor is an Hsp70-2 Regulated Guardian of Lysosomal Stability in Human Cancer. Cancer Res (2007) 67(6):2559. doi: 10.1158/0008-5472.CAN-06-4121 17363574

[74] Bivik C Rosdahl I Ollinger K. Hsp70 Protects Against UVB Induced Apoptosis by Preventing Release of Cathepsins and Cytochrome C in Human Melanocytes. Carcinogenesis (2007) 28(3):537–44. doi: 10.1093/carcin/bgl152 16950797

[75] Lim S Cho HY Kim DG Roh Y Son SY Mushtaq AU. Targeting the Interaction of AIMP2-DX2 With HSP70 Suppresses Cancer Development. Nat Chem Biol (2019 16:31–41. doi: 10.1038/s41589-019-0415-2 31792442

[76] Han JM Park B-J Park SG Oh YS Choi SJ Lee SW. AIMP2/P38, the Scaffold for the Multi-Trna Synthetase Complex, Responds to Genotoxic Stresses via P53. Proc Natl Acad Sci (2008) 105(32):11206. doi: 10.1073/pnas.0800297105 18695251 PMC2516205

[77] Huang LE Bunn HF. Hypoxia-Inducible Factor and its Biomedical Relevance. J Biol Chem (2003) 278(22):19575–8. doi: 10.1074/jbc.R200030200 12639949

[78] Zhou J Schmid T Frank R Brune B. PI3K/Akt is Required for Heat Shock Proteins to Protect Hypoxia-Inducible Factor 1alpha From Pvhl-Independent Degradation. J Biol Chem (2004) 279(14):13506–13. doi: 10.1074/jbc.M310164200 14726529

[79] Kluger HM Chelouche Lev D Kluger Y McCarthy MM Kiriakova G Camp RL. Using a Xenograft Model of Human Breast Cancer Metastasis to Find Genes Associated With Clinically Aggressive Disease. Cancer Res (2005) 65(13):5578–87. doi: 10.1158/0008-5472.CAN-05-0108 15994930

[80] Li H Li Y Liu D Sun H Su D Yang F. Extracellular HSP70/HSP70-Pcs Promote Epithelial-Mesenchymal Transition of Hepatocarcinoma Cells. PloS One (2013) 8(12):e84759. doi: 10.1371/journal.pone.0084759 24386414 PMC3874008

[81] Kasioumi P Vrazeli P Vezyraki P Zerikiotis S Katsouras C Damalas A. Hsp70 (HSP70A1A) Downregulation Enhances the Metastatic Ability of Cancer Cells. Int J Oncol (2019) 54(3):821–32. doi: 10.3892/ijo.2018.4666 PMC6365026 30569142

[82] Sossey-Alaoui K Li X Ranalli TA Cowell JK. WAVE3-Mediated Cell Migration and Lamellipodia Formation are Regulated Downstream of Phosphatidylinositol 3-Kinase. J Biol Chem (2005) 280(23):21748–55. doi: 10.1074/jbc.M500503200 15826941

[83] Sossey-Alaoui K Safina A Li X Vaughan MM Hicks DG Bakin AV. Down-Regulation of WAVE3, a Metastasis Promoter Gene, Inhibits Invasion and Metastasis of Breast Cancer Cells. Am J Pathol (2007) 170(6):2112–21. doi: 10.2353/ajpath.2007.060975 PMC1899429 17525277

[84] Teng Y Ren MQ Cheney R Sharma S Cowell JK. Inactivation of the WASF3 Gene in Prostate Cancer Cells Leads to Suppression of Tumorigenicity and Metastases. Br J Cancer (2010) 103(7):1066–75. doi: 10.1038/sj.bjc.6605850 PMC2965863 20717117

[85] Blachere NE Li Z Chandawarkar RY Suto R Jaikaria NS Basu S. Heat Shock Protein-Peptide Complexes, Reconstituted In Vitro, Elicit Peptide-Specific Cytotoxic T Lymphocyte Response and Tumor Immunity. J Exp Med (1997) 186(8):1315–22. doi: 10.1084/jem.186.8.1315 PMC2199095 9334371

[86] Udono H Srivastava PK. Comparison of Tumor-Specific Immunogenicities of Stress-Induced Proteins Gp96, Hsp90, and Hsp70. J Immunol (1994) 152(11):5398. 8189059

[87] Weng D Calderwood SK Gong J. Preparation of a Heat-Shock Protein 70-Based Vaccine From DC-Tumor Fusion Cells. Methods Mol Biol (2011) 787:255–65. doi: 10.1007/978-1-61779-295-3_19 PMC4088345 21898241

[88] Multhoff G Botzler C Jennen L Schmidt J Ellwart J Issels R. Heat Shock Protein 72 on Tumor Cells: A Recognition Structure for Natural Killer Cells. J Immunol (1997) 158(9):4341. 9126997

[89] Multhoff G Pfister K Gehrmann M Hantschel M Gross C Hafner M. A 14-Mer Hsp70 Peptide Stimulates Natural Killer (NK) Cell Activity. Cell Stress Chaperones (2001) 6(4):337–44. doi: 10.1379/1466-1268(2001)006<0337:AMHPSN>2.0.CO;2 PMC434416 11795470

[90] Stangl S Gross C Pockley AG Asea AA Multhoff G. Influence of Hsp70 and HLA-E on the Killing of Leukemic Blasts by Cytokine/Hsp70 Peptide-Activated Human Natural Killer (NK) Cells. Cell Stress Chaperones (2008) 13(2):221–30. doi: 10.1007/s12192-007-0008-y PMC2673894 18759005

[91] Hromadnikova I Li S Kotlabova K Dickinson AM. Influence of In Vitro IL-2 or IL-15 Alone or in Combination With Hsp 70 Derived 14-Mer Peptide (TKD) on the Expression of NK Cell Activatory and Inhibitory Receptors on Peripheral Blood T Cells, B Cells and NKT Cells. PloS One (2016) 11(3):e0151535–e0151535. doi: 10.1371/journal.pone.0151535 26982331 PMC4794217

[92] Multhoff G Seier S Stangl S Sievert W Shevtsov M Werner C. Targeted Natural Killer Cell–Based Adoptive Immunotherapy for the Treatment of Patients With NSCLC After Radiochemotherapy: A Randomized Phase II Clinical Trial. Clin Cancer Res (2020) 26(20):5368. doi: 10.1158/1078-0432.CCR-20-1141 32873573

[93] Bonam SR Ruff M Muller S. HSPA8/HSC70 in Immune Disorders: A Molecular Rheostat That Adjusts Chaperone-Mediated Autophagy Substrates. Cells (2019) 8(8):849. doi: 10.3390/cells8080849 31394830 PMC6721745

[94] Sahu R Kaushik S Clement CC Cannizzo ES Scharf B Follenzi A. Microautophagy of Cytosolic Proteins by Late Endosomes. Dev Cell (2011) 20(1):131–9. doi: 10.1016/j.devcel.2010.12.003 PMC3025279 21238931

[95] Arndt V Dick N Tawo R Dreiseidler M Wenzel D Hesse M. Chaperone-Assisted Selective Autophagy is Essential for Muscle Maintenance. Curr Biol (2010) 20(2):143–8. doi: 10.1016/j.cub.2009.11.022 20060297

[96] Li Y Xue Y Xu X Wang G Liu Y Wu H. A Mitochondrial FUNDC1/HSC70 Interaction Organizes the Proteostatic Stress Response at the Risk of Cell Morbidity. EMBO J (2019) 38(3):e98786. doi: 10.15252/embj.201798786 30591555 PMC6356068

[97] Tanaka M Mun S Harada A Ohkawa Y Inagaki A Sano S. Hsc70 Contributes to Cancer Cell Survival by Preventing Rab1A Degradation Under Stress Conditions. PloS One (2014) 9(5):e96785–5. doi: 10.1371/journal.pone.0096785 PMC4011886 24801886

[98] Vila-Carriles WH Zhou Z-H Bubien JK Fuller CM Benos DJ. Participation of the Chaperone Hsc70 in the Trafficking and Functional Expression of ASIC2 in Glioma Cells. J Biol Chem (2007) 282(47):34381–91. doi: 10.1074/jbc.M705354200 17878160

[99] Gaiddon C Lokshin M Ahn J Zhang T Prives C. A Subset of Tumor-Derived Mutant Forms of P53 Down-Regulate P63 and P73 Through a Direct Interaction With the P53 Core Domain. Mol Cell Biol (2001) 21(5):1874–87. doi: 10.1128/MCB.21.5.1874-1887.2001 PMC86759 11238924

[100] Kauppinen KP Duan F Wels JI Manor D. Regulation of the Dbl Proto-Oncogene by Heat Shock Cognate Protein 70 (Hsc70). J Biol Chem (2005) 280(22):21638–44. doi: 10.1074/jbc.M413984200 15802271

[101] Ding Y Song N Liu C He T Zhuo W He X. Heat Shock Cognate 70 Regulates the Translocation and Angiogenic Function of Nucleolin. Arterioscler Thromb Vasc Biol (2012) 32(9):e126–34. doi: 10.1161/ATVBAHA.112.247502 22743058

[102] Bański P Mahboubi H Kodiha M Shrivastava S Kanagaratham C Stochaj U. Nucleolar Targeting of the Chaperone Hsc70 is Regulated by Stress, Cell Signaling, and a Composite Targeting Signal Which is Controlled by Autoinhibition. J Biol Chem (2010) 285(28):21858–67. doi: 10.1074/jbc.M110.117291 PMC2898440 20457599

[103] Wang F Bonam SR Schall N Kuhn L Hammann P Chaloin O. Blocking Nuclear Export of HSPA8 After Heat Shock Stress Severely Alters Cell Survival. Sci Rep (2018) 8(1):16820. doi: 10.1038/s41598-018-34887-6 30429537 PMC6235846

[104] Yi L Liling T. The Critical Roles of HSC70 in Physiological and Pathological Processes. Curr Pharm Des (2014) 20(1):101–7. doi: 10.2174/13816128113199990585 23944377

[105] Liu T Daniels CK Cao S. Comprehensive Review on the HSC70 Functions, Interactions With Related Molecules and Involvement in Clinical Diseases and Therapeutic Potential. Pharmacol Ther (2012) 136(3):354–74. doi: 10.1016/j.pharmthera.2012.08.014 22960394

[106] Sandoval JA Hoelz DJ Woodruff HA Powell RL Jay CL Grosfeld JL. Novel Peptides Secreted From Human Neuroblastoma: Useful Clinical Tools? J Pediatr Surg (2006) 41(1):245–51. doi: 10.1016/j.jpedsurg.2005.10.048 16410142

[107] Shan N Zhou W Zhang S Zhang Y. Identification of HSPA8 as a Candidate Biomarker for Endometrial Carcinoma by Using Itraq-Based Proteomic Analysis. OncoTargets Ther (2016) 9:2169–79. doi: 10.2147/OTT.S97983 PMC4835145 27110132

[108] Sun G Cao Y Guo J Li M Dai Y. Heat Shock Cognate Protein 70 Enhanced Integrin β1 Mediated Invasion in Cancer Cells. Cancer Manage Res (2020) 12:981–91. doi: 10.2147/CMAR.S235791 PMC7023913 32104080

[109] Mizukami S Kajiwara C Ishikawa H Katayama I Yui K Udono H. Both CD4+ and CD8+ T Cell Epitopes Fused to Heat Shock Cognate Protein 70 (Hsc70) can Function to Eradicate Tumors. Cancer Sci (2008) 99(5):1008–15. doi: 10.1111/j.1349-7006.2008.00788.x PMC11160078 18341654

[110] Zhang H Wang W Li Q Huang W. Fusion Protein of Atpase Domain of Hsc70 With TRP2 Acting as a Tumor Vaccine Against B16 Melanoma. Immunol Lett (2006) 105(2):167–73. doi: 10.1016/j.imlet.2006.02.004 16580737

[111] Song HY Dunbar JD Zhang YX Guo D Donner DB. Identification of a Protein With Homology to Hsp90 That Binds the Type 1 Tumor Necrosis Factor Receptor. J Biol Chem (1995) 270(8):3574–81. doi: 10.1074/jbc.270.8.3574 7876093

[112] Altieri DC Stein GS Lian JB Languino LR. TRAP-1, the Mitochondrial Hsp90. Biochim Biophys Acta (BBA) - Mol Cell Res (2012) 1823(3):767–73. doi: 10.1016/j.bbamcr.2011.08.007 PMC3263322 21878357

[113] Chen CF Chen Y Dai K Chen PL Riley DJ Lee WH. A New Member of the Hsp90 Family of Molecular Chaperones Interacts With the Retinoblastoma Protein During Mitosis and After Heat Shock. Mol Cell Biol (1996) 16(9):4691. doi: 10.1128/MCB.16.9.4691 8756626 PMC231469

[114] Masgras I Sanchez-Martin C Colombo G Rasola A. The Chaperone TRAP1 as a Modulator of the Mitochondrial Adaptations in Cancer Cells. Front Oncol (2017) 7(58). doi: 10.3389/fonc.2017.00058 PMC5370238 28405578

[115] Byoung Heon K. TRAP1 Regulation of Mitochondrial Life or Death Decision in Cancer Cells and Mitochondria-Targeted TRAP1 Inhibitors. BMB Rep (2012) 45(1):001–6. doi: 10.5483/bmbrep.2012.45.1.1 22281005

[116] Cechetto JD Gupta RS. Immunoelectron Microscopy Provides Evidence That Tumor Necrosis Factor Receptor-Associated Protein 1 (TRAP-1) is a Mitochondrial Protein Which Also Localizes at Specific Extramitochondrial Sites. Exp Cell Res (2000) 260(1):30–9. doi: 10.1006/excr.2000.4983 11010808

[117] Joshi A Dai L Liu Y Lee J Ghahhari NM Segala G. The Mitochondrial HSP90 Paralog TRAP1 Forms an OXPHOS-Regulated Tetramer and is Involved in Mitochondrial Metabolic Homeostasis. BMC Biol (2020) 18(1):10. doi: 10.1186/s12915-020-0740-7 31987035 PMC6986101

[118] Masuda Y Shima G Aiuchi T Horie M Hori K Nakajo S. Involvement of Tumor Necrosis Factor Receptor-Associated Protein 1 (TRAP1) in Apoptosis Induced by Beta-Hydroxyisovalerylshikonin. J Biol Chem (2004) 279(41):42503–15. doi: 10.1074/jbc.M404256200 15292218

[119] Hua G Zhang Q Fan Z. Heat Shock Protein 75 (TRAP1) Antagonizes Reactive Oxygen Species Generation and Protects Cells From Granzyme M-Mediated Apoptosis. J Biol Chem (2007) 282(28):20553–60. doi: 10.1074/jbc.M703196200 17513296

[120] Sciacovelli M Guzzo G Morello V Frezza C Zheng L Nannini N. The Mitochondrial Chaperone TRAP1 Promotes Neoplastic Growth by Inhibiting Succinate Dehydrogenase. Cell Metab (2013) 17(6):988–99. doi: 10.1016/j.cmet.2013.04.019 PMC3677096 23747254

[121] Chen X-S Li L-y Guan Y-d Yang J-m Cheng Y. Anticancer Strategies Based on the Metabolic Profile of Tumor Cells: Therapeutic Targeting of the Warburg Effect. Acta Pharmacol Sin (2016) 37(8):1013–9. doi: 10.1038/aps.2016.47 PMC4973382 27374491

[122] Matassa DS Agliarulo I Avolio R Landriscina M Esposito F. TRAP1 Regulation of Cancer Metabolism: Dual Role as Oncogene or Tumor Suppressor. Genes (2018) 9(4):195. doi: 10.3390/genes9040195 29621137 PMC5924537

[123] Chae YC Angelin A Lisanti S Kossenkov AV Speicher KD Wang H. Landscape of the Mitochondrial Hsp90 Metabolome in Tumours. Nat Commun (2013) 4:2139–9. doi: 10.1038/ncomms3139 PMC3732457 23842546

[124] Yoshida S Tsutsumi S Muhlebach G Sourbier C Lee M-J Lee S. Molecular Chaperone TRAP1 Regulates a Metabolic Switch Between Mitochondrial Respiration and Aerobic Glycolysis. Proc Natl Acad Sci USA (2013) 110(17):E1604–12. doi: 10.1073/pnas.1220659110 PMC3637790 23564345

[125] Park H-K Hong J-H Oh YT Kim SS Yin J Lee A-J. Interplay Between TRAP1 and Sirtuin-3 Modulates Mitochondrial Respiration and Oxidative Stress to Maintain Stemness of Glioma Stem Cells. Cancer Res (2019) 79(7):1369. doi: 10.1158/0008-5472.CAN-18-2558 30683653

[126] Agliarulo I Matassa DS Amoroso MR Maddalena F Sisinni L Sepe L. TRAP1 Controls Cell Migration of Cancer Cells in Metabolic Stress Conditions: Correlations With AKT/P70s6k Pathways. Biochim Biophys Acta (BBA) - Mol Cell Res (2015) 1853(10, Part A):2570–9. doi: 10.1016/j.bbamcr.2015.05.034 26071104

[127] Costantino E Maddalena F Calise S Piscazzi A Tirino V Fersini A. TRAP1, a Novel Mitochondrial Chaperone Responsible for Multi-Drug Resistance and Protection From Apoptotis in Human Colorectal Carcinoma Cells. Cancer Lett (2009) 279(1):39–46. doi: 10.1016/j.canlet.2009.01.018 19217207

[128] Kaul SC Taira K Pereira-Smith OM Wadhwa R. Mortalin: Present and Prospective. Exp Gerontol (2002) 37(10-11):1157–64. doi: 10.1016/S0531-5565(02)00135-3 12470827

[129] Ryu J Kaul Z Yoon AR Liu Y Yaguchi T Na Y. Identification and Functional Characterization of Nuclear Mortalin in Human Carcinogenesis. J Biol Chem (2014) 289(36):24832–44. doi: 10.1074/jbc.M114.565929 PMC4155653 25012652

[130] Daugaard M Rohde M Jäättelä M. The Heat Shock Protein 70 Family: Highly Homologous Proteins With Overlapping and Distinct Functions. FEBS Lett (2007) 581(19):3702–10. doi: 10.1016/j.febslet.2007.05.039 17544402

[131] Dahlseid JN Lill R Green JM Xu X Qiu Y Pierce SK. PBP74, a New Member of the Mammalian 70-Kda Heat Shock Protein Family, is a Mitochondrial Protein. Mol Biol Cell (1994) 5(11):1265–75. doi: 10.1091/mbc.5.11.1265 PMC301151 7865888

[132] Yun C-O Bhargava P Na Y Lee J-S Ryu J Kaul SC. Relevance of Mortalin to Cancer Cell Stemness and Cancer Therapy. Sci Rep (2017) 7:42016–6. doi: 10.1038/srep42016 PMC5292728 28165047

[133] Wadhwa R Takano S Kaur K Deocaris CC Pereira-Smith OM Reddel RR. Upregulation of Mortalin/Mthsp70/Grp75 Contributes to Human Carcinogenesis. Int J Cancer (2006) 118(12):2973–80. doi: 10.1002/ijc.21773 16425258

[134] Ma Z Izumi H Kanai M Kabuyama Y Ahn NG Fukasawa K. Mortalin Controls Centrosome Duplication via Modulating Centrosomal Localization of P53. Oncogene (2006) 25(39):5377–90. doi: 10.1038/sj.onc.1209543 16619038

[135] Lu W-J Lee NP Kaul SC Lan F Poon RTP Wadhwa R. Induction of Mutant P53-Dependent Apoptosis in Human Hepatocellular Carcinoma by Targeting Stress Protein Mortalin. Int J Cancer (2011) 129(8):1806–14. doi: 10.1002/ijc.25857 21165951

[136] Lu WJ Lee NP Kaul SC Lan F Poon RTP Wadhwa R. Mortalin-P53 Interaction in Cancer Cells is Stress Dependent and Constitutes a Selective Target for Cancer Therapy. Cell Death Different (2011) 18(6):1046–56. doi: 10.1038/cdd.2010.177 PMC3131943 21233847

[137] Kaul SC Aida S Yaguchi T Kaur K Wadhwa R. Activation of Wild Type P53 Function by its Mortalin-Binding, Cytoplasmically Localizing Carboxyl Terminus Peptides. J Biol Chem (2005) 280(47):39373–9. doi: 10.1074/jbc.M500022200 16176931

[138] Mylonis I Kourti M Samiotaki M Panayotou G Simos G. Mortalin-Mediated and ERK-Controlled Targeting of HIF-1α to Mitochondria Confers Resistance to Apoptosis Under Hypoxia. J Cell Sci (2017) 130(2):466–79. doi: 10.1242/jcs.195339 27909249

[139] Na Y Kaul SC Ryu J Lee J-S Ahn HM Kaul Z. Stress Chaperone Mortalin Contributes to Epithelial-to-Mesenchymal Transition and Cancer Metastasis. Cancer Res (2016) 76(9):2754. doi: 10.1158/0008-5472.CAN-15-2704 26960973

[140] Li S Lv M Qiu S Meng J Liu W. Nf-κb P65 Promotes Ovarian Cancer Cell Proliferation and Migration via Regulating Mortalin. J Cell Mol Med (2019) 23(6):4338–48. doi: 10.1111/jcmm.14325 PMC6533498 30983127

[141] Kaul SC Deocaris CC Wadhwa R. Three Faces of Mortalin: A Housekeeper, Guardian and Killer. Exp Gerontol (2007) 42(4):263–74. doi: 10.1016/j.exger.2006.10.020 17188442

[142] Yaguchi T Aida S Kaul SC Wadhwa R. Involvement of Mortalin in Cellular Senescence From the Perspective of its Mitochondrial Import, Chaperone, and Oxidative Stress Management Functions. Ann New York Acad Sci (2007) 1100(1):306–11. doi: 10.1196/annals.1395.032 17460192

[143] Custer CD Kaul SC Wadhwa R. On the Brotherhood of the Mitochondrial Chaperones Mortalin and Heat Shock Protein 60. Cell Stress Chaperones (2006) 11(2):116–28. doi: 10.1379/csc-144r.1 PMC1484513 16817317

[144] Wadhwa R Takano S Kaur K Aida S Yaguchi T Kaul Z. Identification and Characterization of Molecular Interactions Between Mortalin/Mthsp70 and HSP60. Biochem J (2005) 391(Pt 2):185–90. doi: 10.1042/BJ20050861 PMC1276915 15957980

[145] Starenki D Hong SK Lloyd RV Park JI. Mortalin (GRP75/HSPA9) Upregulation Promotes Survival and Proliferation of Medullary Thyroid Carcinoma Cells. Oncogene (2015) 34(35):4624–34. doi: 10.1038/onc.2014.392 PMC4451452 25435367

[146] Marzec M Eletto D Argon Y. GRP94: An HSP90-Like Protein Specialized for Protein Folding and Quality Control in the Endoplasmic Reticulum. Biochim Biophys Acta (2012) 1823(3):774–87. doi: 10.1016/j.bbamcr.2011.10.013 PMC3443595 22079671

[147] Munro S Pelham HRB. A C-Terminal Signal Prevents Secretion of Luminal ER Proteins. Cell (1987) 48(5):899–907. doi: 10.1016/0092-8674(87)90086-9 3545499

[148] Altmeyer A Maki RG Feldweg AM Heike M Protopopov VP Masur SK. Tumor-Specific Cell Surface Expression of the -KDEL Containing Endoplasmic Reticular Heat Shock Protein Gp96. Int J Cancer (1996) 69(4):340–9. doi: 10.1002/(SICI)1097-0215(19960822)69:4<340::AID-IJC18>3.0.CO;2-9 8797880

[149] Wiest DL Burgess WH McKean D Kearse KP Singer A. The Molecular Chaperone Calnexin is Expressed on the Surface of Immature Thymocytes in Association With Clonotype-Independent CD3 Complexes. EMBO J (1995) 14(14):3425–33. doi: 10.1002/j.1460-2075.1995.tb07348.x PMC394409 7628443

[150] Subjeck JR Shyy TT. Stress Protein Systems of Mammalian Cells. Am J Physiol-Cell Physiol (1986) 250(1):C1–C17. doi: 10.1152/ajpcell.1986.250.1.C1 3510555

[151] Wiest DL Burkhardt JK Hester S Hortsch M Meyer DI Argon Y. Membrane Biogenesis During B Cell Differentiation: Most Endoplasmic Reticulum Proteins are Expressed Coordinately. J Cell Biol (1990) 110(5):1501–11. doi: 10.1083/jcb.110.5.1501 PMC2200180 2335560

[152] Gass JN Gifford NM Brewer JW. Activation of an Unfolded Protein Response During Differentiation of Antibody-Secreting B Cells. J Biol Chem (2002) 277(50):49047–54. doi: 10.1074/jbc.M205011200 12374812

[153] Lee AS. Mammalian Stress Response: Induction of the Glucose-Regulated Protein Family. Curr Opin Cell Biol (1992) 4(2):267–73. doi: 10.1016/0955-0674(92)90042-B 1599691

[154] Paris S Denis H Delaive E Dieu M Dumont V Ninane N. Up-Regulation of 94-Kda Glucose-Regulated Protein by Hypoxia-Inducible Factor-1 in Human Endothelial Cells in Response to Hypoxia. FEBS Lett (2005) 579(1):105–14. doi: 10.1016/j.febslet.2004.11.055 15620698

[155] Brewer JW Diehl JA. PERK Mediates Cell-Cycle Exit During the Mammalian Unfolded Protein Response. Proc Natl Acad Sci USA (2000) 97(23):12625–30. doi: 10.1073/pnas.220247197 PMC18814 11035797

[156] Nakagawa T Zhu H Morishima N Li E Xu J Yankner BA. Caspase-12 Mediates Endoplasmic-Reticulum-Specific Apoptosis and Cytotoxicity by Amyloid-β. Nature (2000) 403(6765):98–103. doi: 10.1038/47513 10638761

[157] Melnick J Dul JL Argon Y. Sequential Interaction of the Chaperones Bip and GRP94 With Immunoglobulin Chains in the Endoplasmic Reticulum. Nature (1994) 370(6488):373–5. doi: 10.1038/370373a0 7913987

[158] Melnick J Argon Y. Molecular Chaperones and the Biosynthesis of Antigen Receptors. Immunol Today (1995) 16(5):243–50. doi: 10.1016/0167-5699(95)80167-7 7779255

[159] Wu BX Hong F Zhang Y Ansa-Addo E Li Z. Chapter Seven - GRP94/Gp96 in Cancer: Biology, Structure, Immunology, and Drug Development. In: Isaacs J Whitesell L, editors. Advances in Cancer Research. Academic Press (2016). p. 165–90. 10.1016/bs.acr.2015.09.001 26916005

[160] Zhang Y Ansa-Addo E Li Z. GP96: Safeguarding Treg. Oncotarget (2015) 6(24):19936–7. doi: 10.18632/oncotarget.4582 PMC4652964 26164084

[161] Zhang Y Wu BX Metelli A Thaxton JE Hong F Rachidi S. GP96 is a GARP Chaperone and Controls Regulatory T Cell Functions. J Clin Invest (2015) 125(2):859–69. doi: 10.1172/JCI79014 PMC4319419 25607841

[162] Melendez K Wallen ES Edwards BS Mobarak CD Bear DG Moseley PL. Heat Shock Protein 70 and Glycoprotein 96 are Differentially Expressed on the Surface of Malignant and Nonmalignant Breast Cells. Cell Stress Chaperones (2006) 11(4):334–42. doi: 10.1379/CSC-187.1 PMC1712681 17278882

[163] Zheng H Dai J Stoilova D Li Z. Cell Surface Targeting of Heat Shock Protein Gp96 Induces Dendritic Cell Maturation and Antitumor Immunity. J Immunol (2001) 167(12):6731. doi: 10.4049/jimmunol.167.12.6731 11739487

[164] Wanderling S Simen BB Ostrovsky O Ahmed NT Vogen SM Gidalevitz T. GRP94 is Essential for Mesoderm Induction and Muscle Development Because it Regulates Insulin-Like Growth Factor Secretion. Mol Biol Cell (2007) 18(10):3764–75. doi: 10.1091/mbc.e07-03-0275 PMC1995707 17634284

[165] Hua Y White-Gilbertson S Kellner J Rachidi S Usmani SZ Chiosis G. Molecular Chaperone Gp96 is a Novel Therapeutic Target of Multiple Myeloma. Clin Cancer Res (2013) 19(22):6242. doi: 10.1158/1078-0432.CCR-13-2083 24077352 PMC3851294

[166] Patel PD Yan P Seidler PM Patel HJ Sun W Yang C. Paralog-Selective Hsp90 Inhibitors Define Tumor-Specific Regulation of HER2. Nat Chem Biol (2013) 9(11):677–84. doi: 10.1038/nchembio.1335 PMC3982621 23995768

[167] Li X Sun L Hou J Gui M Ying J Zhao H. Cell Membrane Gp96 Facilitates HER2 Dimerization and Serves as a Novel Target in Breast Cancer. Int J Cancer (2015) 137(3):512–24. doi: 10.1002/ijc.29405 25546612

[168] Sabbatino F Favoino E Wang Y Wang X Villani V Cai L. Grp94-Specific Monoclonal Antibody to Counteract BRAF Inhibitor Resistance in BRAF V600E Melanoma. J Trans Med (2015) 13(1):K12. doi: 10.1186/1479-5876-13-S1-K12

[169] Zhang Y Liu R Ni M Gill P Lee AS. Cell Surface Relocalization of the Endoplasmic Reticulum Chaperone and Unfolded Protein Response Regulator GRP78/Bip. J Biol Chem (2010) 285(20):15065–75. doi: 10.1074/jbc.M109.087445 PMC2865300 20208072

[170] Lee AS. The ER Chaperone and Signaling Regulator GRP78/Bip as a Monitor of Endoplasmic Reticulum Stress. Methods (2005) 35(4):373–81. doi: 10.1016/j.ymeth.2004.10.010 15804610

[171] Casas C. GRP78 at the Centre of the Stage in Cancer and Neuroprotection. Front Neurosci (2017) 11(177). doi: 10.3389/fnins.2017.00177 PMC5380735 28424579

[172] Munro S Pelham HRB. An Hsp70-Like Protein in the ER: Identity With the 78 Kd Glucose-Regulated Protein and Immunoglobulin Heavy Chain Binding Protein. Cell (1986) 46(2):291–300. doi: 10.1016/0092-8674(86)90746-4 3087629

[173] Li J Ni M Lee B Barron E Hinton DR Lee AS. The Unfolded Protein Response Regulator GRP78/Bip is Required for Endoplasmic Reticulum Integrity and Stress-Induced Autophagy in Mammalian Cells. Cell Death Different (2008) 15(9):1460–71. doi: 10.1038/cdd.2008.81 PMC2758056 18551133

[174] Cook KL Clarke R. Heat Shock 70 Kda Protein 5/Glucose-Regulated Protein 78 “Amp”Ing Up Autophagy. Autophagy (2012) 8(12):1827–9. doi: 10.4161/auto.21765 PMC3541293 22931685

[175] Cook KL Shajahan AN Wärri A Jin L Hilakivi-Clarke LA Clarke R. Glucose-Regulated Protein 78 Controls Cross-Talk Between Apoptosis and Autophagy to Determine Antiestrogen Responsiveness. Cancer Res (2012) 72(13):3337–49. doi: 10.1158/0008-5472.CAN-12-0269 PMC3576872 22752300

[176] Li Z Wang Y Newton IP Zhang L Ji P Li Z. GRP78 is Implicated in the Modulation of Tumor Aerobic Glycolysis by Promoting Autophagic Degradation of Ikkβ. Cell Signal (2015) 27(6):1237–45. doi: 10.1016/j.cellsig.2015.02.030 25748049

[177] Cha-Molstad H Yu JE Lee SH Kim JG Sung KS Hwang J. Modulation of SQSTM1/P62 Activity by N-Terminal Arginylation of the Endoplasmic Reticulum Chaperone HSPA5/GRP78/Bip. Autophagy (2016) 12(2):426–8. doi: 10.1080/15548627.2015.1126047 PMC4835953 26797053

[178] Cha-Molstad H Sung KS Hwang J Kim KA Yu JE Yoo YD. Amino-Terminal Arginylation Targets Endoplasmic Reticulum Chaperone Bip for Autophagy Through P62 Binding. Nat Cell Biol (2015) 17(7):917–29. doi: 10.1038/ncb3177 PMC4490096 26075355

[179] Abdel Malek MAY Jagannathan S Malek E Sayed DM Elgammal SA Abd El-Azeem HG. Molecular Chaperone GRP78 Enhances Aggresome Delivery to Autophagosomes to Promote Drug Resistance in Multiple Myeloma. Oncotarget (2015) 6(5):3098–110. doi: 10.18632/oncotarget.3075 PMC4413640 25605012

[180] Lee JH Yoon YM Lee SH. GRP78 Regulates Apoptosis, Cell Survival and Proliferation in 5-Fluorouracil-Resistant SNUC5 Colon Cancer Cells. Anticancer Res (2017) 37(9):4943. doi: 10.21873/anticanres.11904 28870916

[181] Sun F-C Wei S Li C-W Chang Y-S Chao C-C Lai Y-K. Localization of GRP78 to Mitochondria Under the Unfolded Protein Response. Biochem J (2006) 396(1):31–9. doi: 10.1042/BJ20051916 PMC1450007 16433633

[182] Hayashi T Su T-P. Sigma-1 Receptor Chaperones at the ER- Mitochondrion Interface Regulate Ca2+ Signaling and Cell Survival. Cell (2007) 131(3):596–610. doi: 10.1016/j.cell.2007.08.036 17981125

[183] Ni M Zhou H Wey S Baumeister P Lee AS. Regulation of PERK Signaling and Leukemic Cell Survival by a Novel Cytosolic Isoform of the UPR Regulator GRP78/Bip. PloS One (2009) 4(8):e6868. doi: 10.1371/journal.pone.0006868 19718440 PMC2729930

[184] Duriez M Rossignol J-M Sitterlin D. The Hepatitis B Virus Precore Protein is Retrotransported From Endoplasmic Reticulum (ER) to Cytosol Through the ER-Associated Degradation Pathway. J Biol Chem (2008) 283(47):32352–60. doi: 10.1074/jbc.M807178200 18805786

[185] Wang X Olberding KE White C Li C. Bcl-2 Proteins Regulate ER Membrane Permeability to Luminal Proteins During ER Stress-Induced Apoptosis. Cell Death Different (2011) 18(1):38–47. doi: 10.1038/cdd.2010.68 PMC2947581 20539308

[186] Ni M Zhang Y Lee AS. Beyond the Endoplasmic Reticulum: Atypical GRP78 in Cell Viability, Signalling and Therapeutic Targeting. Biochem J (2011) 434(2):181–8. doi: 10.1042/BJ20101569 PMC3353658 21309747

[187] Reddy RK Mao C Baumeister P Austin RC Kaufman RJ Lee AS. Endoplasmic Reticulum Chaperone Protein GRP78 Protects Cells From Apoptosis Induced by Topoisomerase Inhibitors: ROLE of ATP BINDING SITE in SUPPRESSION of CASPASE-7 ACTIVATION. J Biol Chem (2003) 278(23):20915–24. doi: 10.1074/jbc.M212328200 12665508

[188] Morris JA Dorner AJ Edwards CA Hendershot LM Kaufman RJ. Immunoglobulin Binding Protein (Bip) Function is Required to Protect Cells From Endoplasmic Reticulum Stress But is Not Required for the Secretion of Selective Proteins. J Biol Chem (1997) 272(7):4327–34. doi: 10.1074/jbc.272.7.4327 9020152

[189] Matsumoto A Hanawalt PC. Histone H3 and Heat Shock Protein GRP78 are Selectively Cross-Linked to DNA by Photoactivated Gilvocarcin V in Human Fibroblasts. Cancer Res (2000) 60(14):3921. 10919670

[190] Zhai L Zhai L Kita K Wano C Wu Y Sugaya S Suzuki N. Decreased Cell Survival and DNA Repair Capacity After UVC Irradiation in Association With Down-Regulation of GRP78/Bip in Human Rsa Cells. Exp Cell Res (2005) 305(2):244–52. doi: 10.1016/j.yexcr.2005.01.002 15817150

[191] Jianze L Amy SL. Stress Induction of GRP78/Bip and its Role in Cancer. Curr Mol Med (2006) 6(1):45–54. doi: 10.2174/156652406775574523 16472112

[192] Lee AS. GRP78 Induction in Cancer: Therapeutic and Prognostic Implications. Cancer Res (2007) 67(8):3496. doi: 10.1158/0008-5472.CAN-07-0325 17440054

[193] Fu Y Wey S Wang M Ye R Liao C-P Roy-Burman P. Pten Null Prostate Tumorigenesis and AKT Activation are Blocked by Targeted Knockout of ER Chaperone GRP78/Bip in Prostate Epithelium. Proc Natl Acad Sci USA (2008) 105(49):19444–9. doi: 10.1073/pnas.0807691105 PMC2614780 19033462

[194] Misra UK Pizzo SV. Modulation of the Unfolded Protein Response in Prostate Cancer Cells by Antibody-Directed Against the Carboxyl-Terminal Domain of GRP78. Apoptosis (2010) 15(2):173–82. doi: 10.1007/s10495-009-0430-y 20091233

[195] Cook KL Soto-Pantoja DR Clarke PAG Cruz MI Zwart A Wärri A. Endoplasmic Reticulum Stress Protein GRP78 Modulates Lipid Metabolism to Control Drug Sensitivity and Antitumor Immunity in Breast Cancer. Cancer Res (2016) 76(19):5657–70. doi: 10.1158/0008-5472.CAN-15-2616 PMC5117832 27698188

[196] Cook KL Soto-Pantoja DR. “Upregulation” of CD47 by the Endoplasmic Reticulum Stress Pathway Controls Anti-Tumor Immune Responses. biomark Res (2017) 5(1):26. doi: 10.1186/s40364-017-0105-8 28815041 PMC5557514

[197] Madden E Logue SE Healy SJ Manie S Samali A. The Role of the Unfolded Protein Response in Cancer Progression: From Oncogenesis to Chemoresistance. Biol Cell (2019) 111(1):1–17. doi: 10.1111/boc.201800050 30302777

[198] Rao RV Peel A Logvinova A del Rio G Hermel E Yokota T. Coupling Endoplasmic Reticulum Stress to the Cell Death Program: Role of the ER Chaperone GRP78. FEBS Lett (2002) 514(2-3):122–8. doi: 10.1016/S0014-5793(02)02289-5 PMC3971841 11943137

[199] Dauer P Sharma NS Gupta VK Durden B Hadad R Banerjee S. ER Stress Sensor, Glucose Regulatory Protein 78 (GRP78) Regulates Redox Status in Pancreatic Cancer Thereby Maintaining “Stemness”. Cell Death Dis (2019) 10(2):132. doi: 10.1038/s41419-019-1408-5 30755605 PMC6372649

[200] Chang C-W Chen Y-S Tsay Y-G Han C-L Chen Y-J Yang C-C. ROS-Independent ER Stress-Mediated NRF2 Activation Promotes Warburg Effect to Maintain Stemness-Associated Properties of Cancer-Initiating Cells. Cell Death Dis (2018) 9(2):194. doi: 10.1038/s41419-017-0250-x 29416012 PMC5833380

[201] Agarwal E Altman BJ Seo JH Ghosh JC Kossenkov AV Tang H-Y. Myc-Mediated Transcriptional Regulation of the Mitochondrial Chaperone TRAP1 Controls Primary and Metastatic Tumor Growth. J Biol Chem (2019) 294(27):10407–14. doi: 10.1074/jbc.AC119.008656 PMC6615691 31097545

[202] Zavareh RB Spangenberg SH Woods A Martínez-Peña F Lairson LL. HSP90 Inhibition Enhances Cancer Immunotherapy by Modulating the Surface Expr*ession of Multiple Immune Checkpoint Proteins* . Cell Chem Biol (2021) 28(2):158–168.e5. doi: 10.1016/j.chembiol.2020.10.005 33113406

[203] Zappasodi R Ruggiero G Guarnotta C Tortoreto M Tringali C Cavanè A. HSPH1 Inhibition Downregulates Bcl-6 and C-Myc and Hampers the Growth of Human Aggressive B-Cell non-Hodgkin Lymphoma. Blood (2015) 125(11):1768–71. doi: 10.1182/blood-2014-07-590034 25573990

