# Corrigendum: HSP70 and HSP90 in cancer: cytosolic, endoplasmic reticulum and mitochondrial chaperones of tumorigenesis

**DOI:** 10.3389/fonc.2023.1210051

**Published:** 2023-05-03

**Authors:** Zarema Albakova, Yana Mangasarova, Akhmet Albakov, Liliya Gorenkova

**Affiliations:** ^1^ Department of Biology, Lomonosov Moscow State University, Moscow, Russia; ^2^ Chokan Limited Liability Partnership (LLP), Almaty, Kazakhstan; ^3^ National Research Center for Hematology, Moscow, Russia; ^4^ Department of Innovation, Almaty, Kazakhstan

**Keywords:** heat shock proteins, HSP70, HSP90, GRP78, GRP94, TRAP1, mortalin, cancer

**Additional affiliation(s)**

In the published article, there was an error regarding the affiliation(s) for Zarema Albakova. As well as having affiliation 1, they should also have the following affiliation - Chokan Limited Liability Partnership (LLP), Almaty, Kazakhstan.

The authors apologize for this error and state that this does not change the scientific conclusions of the article in any way. The original article has been updated.

